# Unbiased profiling of translational landscape reveals TNFR2 as a translation-dependent vulnerability in colorectal cancer

**DOI:** 10.1186/s13046-026-03706-6

**Published:** 2026-04-11

**Authors:** Yiying Chen, Yufeng Gao, Haixia Wang, Weiqian Li, Fanqi Zhou, Hao Sun, Jia Yu, Dongling Zou, Fang Wang

**Affiliations:** 1 State Key Laboratory of Medical Molecular Biology, Department of Biochemistry and Molecular Biology, Haihe Laboratory of Cell Ecosystem, Institute of Basic Medical Sciences, School of Basic Medicine, Chinese Academy of Medical Sciences, Peking Union Medical College, Beijing, China; 2 The Key Laboratory of RNA and Hematopoietic Regulation, Chinese Academy of Medical Sciences, Beijing, China; 3 Clinical Biobank, Peking Union Medical College Hospital, Chinese Academy of Medical Sciences & Peking Union Medical College, Beijing, China; 4 Department of Gynecologic Oncology, Chongqing University Cancer Hospital & Chongqing Cancer Institute & Chongqing Cancer Hospital, Chongqing, China; 5 Chongqing Specialized Medical Research Center of Ovarian Cancer, Chongqing, China; 6 Organoid Transformational Research Center, Chongqing Key Laboratory of Translational Research for Cancer Metastasis and Individualized Treatment, Chongqing University Cancer Hospital, Chongqing, China; 7 Department of Gastrointestinal Oncology, Chongqing University Cancer Hospital & Chongqing Cancer Institute & Chongqing Cancer Hospital, Chongqing, China

**Keywords:** Colorectal cancer, Translatome, Translational dysregulation, TNFR2, Therapeutic target

## Abstract

**Background:**

Translational dysregulation plays a key role in tumour initiation and progression, including in colorectal cancer (CRC). However, the mechanisms underlying translational control and the systematic characterization of translation-dependent vulnerabilities remain poorly understood in CRC.

**Methods:**

We profiled 34 colorectal tissue samples to define a comprehensive translatomic landscape of CRC, uncovering a set of unannotated ORFs from coding (both in-frame and out-of-frame) and long noncoding RNAs (lncRNAs). Additionally, we delineated five translatomic clusters that significantly correlated with specific clinical features and common CRC mutations.

**Results:**

Further analysis revealed a functionally coherent network of 449 genes that are exclusively regulated at the translational level and specifically promote CRC progression by modulating cell growth and immune responses. Among these, tumour necrosis factor receptor 2 (*TNFR2*) stood out as the most promising vulnerability with significant translational activation in CRC. Pharmacological inhibition of *TNFR2* suppressed tumorigenesis in both cell-based and organoid models, and was further validated in vivo using patient-derived organoid xenograft (PDOX) models.

**Conclusion:**

Our study establishes a systematic framework bridging unbiased translatome discovery to functional validation, in which we identified and functionally validated the therapeutic vulnerability of translationally activated *TNFR2* in CRC.

**Supplementary Information:**

The online version contains supplementary material available at 10.1186/s13046-026-03706-6.

## Background

Colorectal cancer (CRC) is one of the leading causes of cancer-related death worldwide; it is often diagnosed at a late stage, resulting in poor prognosis [1, 2]. Understanding CRC tumorigenesis is essential for developing effective targeted therapies. Many insights into the mechanisms underlying CRC progression have emerged from multiomics studies that delineate functional and regulatory networks [3–5].

The cellular transcriptome (mRNA profile) reflects both transcription and mRNA turnover, but it often shows poor correlation with the quantitative proteome, particularly in multicellular organisms [6, 7]. This underscores the importance of translational regulation [8–11]. In solid tumours, the transcriptome influences key aspects of carcinogenesis and tumour progression, including epithelial–mesenchymal transition (EMT) [12, 13], acquisition and maintenance of tumour stemness [14, 15], transitions between proliferation and dedifferentiation, and the emergence of aggressive, drug-resistant phenotypes [16]. Despite these insights, the role of translational regulation in CRC remains largely underexplored.

In this study, we systematically investigated the translatomes of 34 human tissues including 19 CRC tumour tissues and 15 paracancerous tissues, identifying 5,166 unannotated open reading frames (ORFs). Based on the above multi-omics analysis results, we also identified 449 genes primarily regulated at the translational level, exhibiting differential translation efficiencies. Tumour necrosis factor receptor 2 (*TNFR2*), also known as TNF receptor superfamily member 1B (*TNFRSF1B*), was among these genes and displayed significantly increased translation efficiency in CRC. Subsequent experiments confirmed that targeting TNFR2 disrupted carcinogenesis in both ex vivo and in vivo models. Collectively, our study provides a panoramic atlas of translational dysregulation in CRC and, arising from this resource, systematically nominates and validates TNFR2 as a potential therapeutic target, thereby expanding our understanding of cancer biology and offering a concrete therapeutic opportunity.

## Methods

### Clinical samples collection and classification

36 tissue samples were collected from 21 patients at Chongqing University Cancer Hospital (Chongqing, China) with their prior written informed consent. Of these, 34 tissue specimens were used for sequencing analysis and 9 tissue specimens were used for experimental validation; detailed usage of these specimens can be found in Table S1. This study, conducted in accordance with the Declaration of Helsinki, was approved by the Ethics Committees of the Cancer Institute and Hospital, Chinese Academy of Medical Sciences (approval number SB2024034), and Chongqing University Cancer Hospital (approval number CZLS2020217-A).

In this study, the control tissues used were all “paracancerous tissues”, which were collected from histologically normal-appearing regions within 2 cm of the tumour edge. These tissues were confirmed by a pathologist to be free of pathological abnormalities. “Tumour tissues” were procured from the core of the tumour lesion and pathologically confirmed as carcinoma. Immediately after surgical resection, tissue samples were processed on ice within 15 min. A pathologist dissected the gross specimen to obtain small fragments (approximately 50–100 mg each) from adjacent areas. These fragments were allocated in parallel for different downstream applications (e.g., sequencing, organoid culture, protein analysis) and separately aliquoted. Processing and preservation were performed without delay: one portion was snap-frozen for nucleic acid extraction (sequencing), one was placed in specialized preservation medium for organoid culture, and another was frozen or fixed for subsequent protein validation. This protocol ensured the biological consistency of materials used for different analyses and prevented repeated freeze-thaw cycles.

### Cell lines

HCT116, RKO and HEK293T cells were obtained from ATCC. These cell lines were cultured in IMDM (Cat No. 12440053, Gibco, Thermo Fisher Scientific, Germany) or DMEM (Cat No. 11965092, Gibco, Thermo Fisher Scientific, Germany) supplemented with 10% foetal bovine serum (Cat No. A5256701, Gibco, Thermo Fisher Scientific, Germany) and 1% antibiotic solution (penicillin 10,000 U/ml, streptomycin 100 mg/ml) (Cat No. P7630, Solarbio, Beijing, China). The cells were maintained at 37 °C in an incubator with 5% CO_2_. Cell line authentication: All cell lines (HCT116, RKO) were routinely authenticated using short tandem repeat (STR) profiling and compared with reference profiles from the source database (ATCC). Mycoplasma testing: Cell lines were regularly tested for Mycoplasma contamination via PCR or commercial detection kits prior to use and during routine passaging. Passaging protocol: Cells were passaged using trypsin-EDTA (Cat No. T1320, Solarbio, Beijing, China) when reaching 80–90% confluence, with split ratios adjusted according to growth rates (typically 1:3 to 1:5), and passage numbers were recorded to ensure experimental consistency.

### Plasmids

To construct the shRNA plasmids, complementary sense and antisense oligonucleotides encoding shRNAs targeting TNFR2 were synthesized (shTNFR2-1: 5’-CCGGGCCGGCTCAGAGAATACTATGCTCGAGCATAGTATTCTCTGAGCCGGCTTTTTG 3’, 5’ AATTCAAAAA-GCCGGCTCAGAGAATACTATG-CTCGAG- CATAGTATTCTCTGAGCCGGC 3’; shTNFR2-2: 5’ CCGGGCCAGACCAGGAACTGAAACACTCGAGTGTTTCAGTTCCTGGTCTGGCTTTTTG 3’, 5’AATTCAAAAAGCCAGACCAGGAACTGAAACACTCGAGTGTTTCAGTTCCTGGTCTGGC 3’) and annealed and were then cloned and inserted into the pLKO.1 vector (Cat No. 10878, Addgene, Watertown, USA).

### Lentivirus production

Lentiviruses were packaged in HEK293T cells using a three-plasmid system. Briefly, HEK293T cells cultured to 70–80% confluence (seeded in 10 cm dishes) were co-transfected using the Neofect™ DNA Transfection Reagent (Neofect (Beijing) Biotech Co., Ltd.) with the following three plasmids: the packaging plasmid pMD2.G (expressing the VSV-G envelope glycoprotein), the packaging plasmid psPAX2 (providing viral structural proteins and replicase), and the lentiviral transfer plasmid carrying the gene of interest. Cell culture supernatants containing viral particles were harvested at 48 and 72 h post-transfection. The combined supernatants were filtered through a 0.45 μm filter to remove cell debris and then concentrated by ultracentrifugation (25,000 rpm for 2 h at 4 °C). The concentrated viral particles were resuspended in a small volume of sterile PBS or culture medium, aliquoted, and stored at -80 °C. Viral titers were determined by a functional titer assay based on fluorescence or resistance marker expression after infecting HEK293T cells.

### Lentiviral transduction

A total of 1 × 10^5^ cells were preseeded in the wells of a 6-well plate. When the cell confluence was appropriate (40%-50%), the medium was replaced with 2 mL of fresh complete medium. Lentiviral particles (at a multiplicity of infection of 20) and polybrene (at a final concentration of 6 µg/mL; Cat. No. H8761, Solarbio, Beijing, China) were then added to the medium. After 12 h of incubation, the virus-containing medium was removed and replaced with 2 mL of fresh complete medium.

### Cell proliferation assays

For the cell proliferation assays, RKO, RKO-shTNFR2-1, RKO-shTNFR2-2, HCT116, HCT116-shTNFR2-1 and HCT116-shTNFR2-2 cells (1,000 cells per well) were seeded in 96-well plates. Then, 100 µL of medium and 10 µL of CCK8 (Cat No. CA1210, Solarbio, Beijing, China) solution were added to each well, and the plates were incubated at 37 °C for 4 h. The absorbance at 450 nm was measured with a spectrophotometer.

### Clonogenic assay

First, 1,000 RKO, RKO-shTNFR2-1, RKO-shTNFR2-2, HCT116, HCT116-shTNFR2-1 and HCT116-shTNFR2-2 cells were seeded in each well of a 6-well plate and cultured for 7–14 days. Fixative (Cat No. P1110, Solarbio, Beijing, China) was then added to the cells at room temperature for 15 min and discarded. After washing with PBS, crystal violet ammonium oxalate solution (Cat No. G1062, Solarbio, Beijing, China) was added to each well for 20 min at room temperature. The plate was then thoroughly rinsed with running tap water until the background became clear​ and air-dried. Macroscopic colonies were finally photographed using a standard digital camera or a gel documentation system under consistent lighting conditions.

### DNA extraction and whole-exome sequencing

DNA was extracted from CRC tissues and organoids using DNAzol reagent (Cat No. 10503027, Invitrogen, Carlsbad, CA, USA). The genomic DNA was randomly broken into fragments of 180–280 bp. An Agilent SureSelect Human All Exon V6 kit (Cat No. 5190–8863, Agilent, CA, USA) was used for library construction and capture experiments. After terminal repair, phosphorylation and the addition of an A tail, the fragments were connected with splices at both ends to prepare the DNA library.

### RNA extraction and quantitative real-time reverse transcription PCR (qRT-PCR)

Total RNA was extracted using TRIzol (Cat No. 15596018CN, Invitrogen, Carlsbad, CA, USA). RNA was reverse transcribed to cDNA by using a Reverse Transcription Kit (Cat No. RR036A, Takara, Dalian, China). RNA transcript levels were measured with a Bio-Rad CFX96 system (Bio-Rad, Foster City, CA, USA) using a SYBR Premix Ex Taq Kit (Cat No. RR039B, Takara, Dalian, China). Expression levels in all samples were normalized to those of GAPDH or 18 S rRNA. The sequences of the primers used for qRT-PCR are shown in Table S2.

### HLA peptide isolation and liquid chromatography–tandem mass spectrometry (LC–MS/MS) analysis

After the frozen tissue was thoroughly ground, cell lysis buffer was added. The mixture was incubated on ice for 60 min prior to 5 min of sonication and high-speed centrifugation to collect the supernatant. The supernatant was then subjected to enrichment and elution of HLA-peptide complexes (HLA-I) using the NEO Discovery HLA-I Peptide Enrichment Kit (Cat No. BZNEO H-I-3, Baizhen Biotechnologies). The HLA-peptide complexes recovered were separated using a reverse-phase column to obtain immunopeptide fractions. The collected fractions were analyzed by LC‒MS/MS. After reconstitution in formic acid-water solution, an appropriate volume of peptide sample was subjected to separation using a Nano-Elute high-performance liquid chromatography system. The immunopeptidome was analyzed with a high-resolution mass spectrometer (Bruker timsTOF Pro2). The instrument parameters used were set as follows: acquisition mode: DDA; total run time: 60 min; full mass scan range: m/z 100-4,000; parallel accumulation–serial fragmentation (PASEF) setting: 10 MS/MS (total cycle time 2.22 s); and ion intensity threshold: 2,500. Then, raw MS files (.d format) were generated.

### Analysis of MS data for HLA peptides

All raw MS data files were analyzed with the PEAKS DeepNovo Peptidome workflow (Bioinformatics Solutions, Inc.) against a database of the human sequences in UniProt/SwissProt merged with a file containing 5,166 unannotated ORF sequences, in which *de novo* sequencing, database searches, and homology searches were combined for peptide identification. On this platform, the SPIDER algorithm is specifically designed to detect peptide mutations, through which sequence variants and mutations that are defined as homologues can be found. The peptides that could not be found in the protein database were defined via DeepNovo, and the average local confidence (ALC%) score of each peptide was selected as > 50%. No enzyme digestion was selected, because HLA peptides are naturally synthesized peptides without artificial enzymatic digestion. The precursor mass and fragment ion mass tolerances were set to 20.0 ppm and 0.05 Da, respectively. Methionine oxidation (15.99 Da) was set as a variable modification, and the false discovery rate (FDR) was set to 1%. Peptides with a length between 7 and 16 amino acids were selected. The DeepImmu™ Neoantigen Discovery platform (Bioinformatics Solutions, Inc.) was used to predict the immunogenicity of the immunopeptides.

### Western blot analysis

Cell samples were fully lysed on ice with lysis buffer, followed by centrifugation at 12,000 × g for 15 min at 4 °C to collect the supernatant (total protein). Equal amounts of protein samples were separated by 10% SDS-PAGE and then transferred to PVDF membranes via wet transfer (transfer condition: constant current of 250 mA in transfer buffer for 90 min). After transfer, the PVDF membranes were blocked with 5% nonfat milk at room temperature for 1 h, followed by incubation with specific primary antibodies (anti-TNFR2, Cat No. 19272-1-AP, anti-DNMT3B, Cat No. 26971-1-AP, anti-RIPK2 Cat No. 15366-1-AP, anti-β-actin, Cat No. 20536-1-AP, all purchased from Proteintech) overnight at 4 °C. On the next day, membranes were washed three times with 1XTBST (Cat No. T1085, Solarbio, Beijing, China) (7 min each), then incubated with HRP-conjugated secondary antibodies (rabbit anti-mouse IgG or mouse anti-rabbit IgG, 1:10,000 diluted in 5% nonfat milk) at room temperature for 1 h. Finally, membranes were washed three times with 1XTBST (Cat No. T1085, Solarbio, Beijing, China) (7 min each) and detected by chemiluminescence imaging.

### Polysome profiling

All tissues from CRC patients were flash frozen and stored in liquid nitrogen. The tissues were lysed in lysis buffer (100 µg/ml cycloheximide (CHX) (Cat No. HY-12320, MCE, NJ, USA), 1% Triton X-100 (Cat No. T8200, Solarbio, Beijing, China), 50 mM HEPES (Cat No. H8090, Solarbio, Beijing, China), 2 mM MgCl_2_ (Cat No. R0971, Solarbio, Beijing, China), 10% glycerine (Cat No. IG3510, Solarbio, Beijing, China), 100 mM KCl (Cat No. IR9022, Solarbio, Beijing, China), 1 mM dithiothreitol (DTT) (Cat No. ID5850, Solarbio, Beijing, China), 20 U/ml RNase Inhibitor (Cat No. N8080119, Invitrogen, Carlsbad, CA, USA), 1Xprotease inhibitor cocktail (Cat No. 78429, Invitrogen, Carlsbad, CA, USA). The tissue lysate was collected and loaded onto a 20/50% w/v sucrose gradient prepared in lysis buffer. The gradient was centrifuged at 27,500 rpm (Beckman, rotor SW41) at 4 °C for 4 h. The samples were then fractionated and analyzed with a Gradient Station (BioCamp) equipped with an ECONO UV monitor (Bio-Rad) and a fraction collector (FC203B, Gilson). The fractions were categorized and used for RNA extraction with TRIzol reagent for subsequent RT-PCR analysis.

### Ribosome profiling

Before the experiment, the tissues (0.1–0.2 g) were cut into pieces. Then, the subsequent steps were performed as described previously [17], with the following modifications. In brief, 500 µl of lysate was used to prepare 50 mg samples. Then, these samples were crushed into powder in a grinder (TKA, T 10 basic ULTRA-TURRAX) filled with lysis buffer (100 µg/ml CHX, 1% Triton X-100, 50 mM HEPES, 2 mM MgCl_2_, 10% glycerine, 100 mM KCl, 1 mM DTT, 20 U/ml RNase inhibitor, protease inhibitor cocktail). A total of 1/5 µl of lysate was used to prepare the total RNA library, and ribosome footprinting and subsequent preparation of the ribosome-protected RNA fragment (RPF) library were performed in parallel with 4/5 µl of lysate. For RPF library preparation, RNase I was added to 400 µl of lysate, and the mixture was incubated for 45 min at room temperature with gentle mixing, followed by chilling on ice. Then, nuclease digestion was stopped by adding 1 µl of RNase inhibitor. RPFs were purified with MicroSpinS-400 columns (GE Healthcare Life Sciences) prior to size selection on a 15% TBE-urea gel, and the 26–34 nt fragment was then excised from the gel. rRNA was depleted using the NEBNext rRNA Depletion Kit (Cat No. E7400L, New England Biolabs, MA, USA). Following end repair and 3’ adaptor ligation, RNA was reverse transcribed using SuperScript III (Cat No. 18080044, Thermo Fisher, USA). cDNA libraries were then prepared as described and sequenced with the Illumina HiSeq X Ten platform.

### Organoid culture

In this study, all samples used for organoid generation and biological analyses were obtained from Chongqing University Cancer Hospital. Fresh tissue samples were kept in ice-cold DMEM/F12 (Cat No. 12634010, Thermo Fisher, USA) until processed. In brief, tissue was cut into small pieces, washed with ice-cold PBS three times and subsequently digested with TrypLE Express (Cat No. 12604013, Thermo Fisher, USA) for 30 min at 37 °C with vigorous pipetting every 5 min. The supernatant was collected and filtered through 70 μm and 40 μm filters. Then, the filtrate was centrifuged at 1,200 rpm at 4 °C for 10 min. The cells were suspended in Matrigel (growth factor reduced; Cat No. 356231, BD Biosciences, CA, USA) and dispensed into 48-well culture plates (25 µl of Matrigel per well).

The culture media used were as follows: advanced DMEM/F12 supplemented with penicillin/streptomycin, 10 mM HEPES, 2 mM GlutaMAX, 1× B27 (Cat No. 17504044, Thermo Fisher Scientific, USA), 1× N2 (Cat No. 17502001, Thermo Fisher Scientific, USA), 10 nM gastrin I (Cat No. 10047-33-3, Sigma‒Aldrich, USA) and 1 mM N-acetylcysteine (Cat No. 38520-57-9, Sigma‒Aldrich, USA). Then, 500 ng/ml R-spondin-1 (Cat No. R-spondin-1, PeproTech, USA), 50 ng/ml human recombinant EGF (Cat No. PHG0311L, PeproTech, USA), 2 µM A83-01 (Cat No. 2939, Tocris, UK), 100 ng/ml human recombinant Noggin (Cat No. 120–10 C, PeproTech, USA), 10 µM SB202190 (Cat No. 152121-30-7, Sigma‒Aldrich, USA) and 10 µM Y-27,632 (Cat No. 129830-38-2, Sigma‒Aldrich, USA) were added. To prevent contamination, Primocin (Cat No. ant-pm-2, InvivoGen, USA) was added to the culture medium. For passaging, the organoids were collected, washed, fragmented, and then digested with TrypLE Express.

For passaging, organoids were washed with 1 mL of HBSS and then dissociated by adding 1 mL of TrypLE Express followed by mechanical pipetting. After incubation at 37 °C for 15 min, the mixture was transferred to a 15 mL centrifuge tube, washed with 10 mL of HBSS, and centrifuged at 1,200 rpm for 4 min. The pelleted organoids were finally embedded in Matrigel at a 1:2 or 1:3 ratio.

### H&E and IHC staining

Organoids and tissues were fixed in freshly prepared 4% paraformaldehyde (Sigma Aldrich). After the sample was embedded, the paraffin block was sliced, and after dewaxing, the sections were subjected to HE staining. Neutral gum was then used to mount the section. IHC staining for Ki-67, CDX2, and CK20 was performed. The sections were subjected to antigen retrieval in EDTA buffer (pH 9.0) and then treated with 3% hydrogen peroxide solution to block endogenous peroxidase activity. After the sections were blocked with 3% BSA, antibodies (anti-Ki67, 1:500, Cat No. GB111499-50, Servicebio; anti-CK20, Cat No. GB112050-50, 1:1,000, Servicebio; anti-CDX2, Cat No. GB150083-100, 1:1,000, Servicebio) were added, and the sections were incubated overnight at 4 °C. The slides were removed and washed with PBS (pH 7.4), and the secondary antibody was then added dropwise for incubation at room temperature for 50 min. After washing with PBS (pH 7.4), colour development was carried out with freshly prepared DAB colour developing solution. The slides were rinsed with running water to terminate colourization. Afterwards, the sections were sequentially counterstained with haematoxylin for approximately 3 min, washed with tap water, differentiated with haematoxylin differentiation solution for a few seconds, rinsed with tap water, destained with haematoxylin decolourization solution, and rinsed with running water. Finally, the sections were dehydrated with alcohol and sealed with neutral gum.

### Drug treatments

For drug treatment, organoids were isolated from Matrigel by Cultrex Organoid Harvesting Solution (R༆D SYSTEMS) and then cultured in 384-well cell culture plates. About 30 ± 10 organoids were seed in each well, covered with 20 µl medium. The final concentration of TNFα (Cat No. HY-P1875, MCE, USA), SIM2457 (Cat No. HY-134836, MCE, USA), SIM0235 (Cat No. SIM1811-03, Simcere Pharmaceutical Group, Jiangsu, China) and IgG1 κ (Cat No. HY-P991195, MCE, USA) were 1 ng/mL, 5 µM, 3 mg/mL and 3 mg/mL, respectively. Each drug concentration contained five duplicate wells respectively.

### Quantification of global m^6^A RNA methylation

The global m^6^A methylation level was assessed using the EpiQuik m^6^A RNA Methylation Quantification Kit (Colorimetric) (Cat No. P-9005, Epigentek, USA) with total RNA isolated as described above. The assay was performed following the manufacturer’s instructions. In brief, 200 ng of total RNA from each sample was used for the assay. To determine the m^6^A content, a standard curve was generated using the serial dilutions of the positive control (with defined m^6^A levels) provided in the kit, run in parallel with the samples. After capturing total RNA and m^6^A on the assay wells, and subsequent incubation with capture and detection antibodies, the signal was developed. The absorbance was measured at 450 nm using a microplate reader. The absorbance value of each sample was then interpolated from the standard curve to calculate the absolute amount of m^6^A (ng). The global m^6^A level was finally expressed as the absolute m^6^A content per 200 ng of input total RNA. Each sample, along with kit controls, was analyzed in triplicate.

### Organoid viability assays

A CellTiter-Glo^®^ (Promega) assay was used to evaluate organoid viability following 3 or 6 days of drug incubation or TNFR2 knockdown. The assay was performed according to the following steps: the plate and reagent were first equilibrated to room temperature (22–25 °C) for about 30 min. Subsequently, a volume of reagent equal to the culture medium volume in each well was added. After vigorous mixing for 5 min to induce cell lysis, the plate was incubated at room temperature for an additional 25 min to stabilize the luminescent signal before recording. Graphpad Prism 10 was used to draw IC50 curve and calculate IC50 value. All screening plates were subjected to stringent quality control measures.

### Organoid measurement

For quantitative assessment of organoid size, the horizontal cross-sectional area (projected surface area) was measured. Multiple non-overlapping bright-field images were randomly captured from each well of a 384-well plate using a Zeiss Axio Observer Z1 inverted microscope equipped with a 10× objective. Image acquisition settings (exposure time, light intensity) were kept constant across all groups within the same experiment. All images were analyzed using ImageJ software (National Institutes of Health). Images were first converted to 8-bit grayscale, and area measurements were performed using a combination of manual tracing (Freehand Selections tool) and an automated workflow. Automated analysis involved applying a uniform threshold (determined based on control wells) to grayscale images to generate a binary mask, separating foreground from background. Organoids were identified using the “Analyze Particles” function, with the size range set according to the manually measured areas of the largest and smallest well-defined organoids in control wells. Circularity parameters (e.g., 0.2–1.0) could be set to exclude irregular objects, and particles touching image borders were automatically excluded. All automated detection results were visually verified by an investigator, and any misidentifications were manually corrected. For each well, approximately 50 organoids were measured by continuously analyzing random fields of view until the target number was reached or most of the well area had been sampled, ensuring representative sampling. To minimize potential statistical bias in quantitative analysis, we randomly captured multiple images from different fields of view within the same well and used the average values as the analytical result. Organoids exhibiting blurred edges due to being out of the focal plane were excluded from the analysis of organoid diameter. Organoids with a diameter below 25 μm were excluded from the quantification of organoid number. After 3 days of culture, the total number of organoids per well was manually counted under a light microscope to assess growth efficiency, as automatic counting was unreliable due to high density and frequent clustering. Finally, all area measurements (µm²) and counts per well were compiled, and the mean area and standard deviation were calculated. These well-level metrics were used for statistical comparisons between groups.

### Animal experiments

Patient-derived CRC organoids from three proliferative cases were harvested using organoid recovery solution and subsequently inoculated into the right axilla of BALB/c-nu athymic nude mice (1 × 10⁶ organoid-derived cells per mouse). The mice for each organoid were randomly divided into three groups (*n* = 5 per group): a blank control group receiving saline, an isotype control group receiving IgG1 κ, and an experimental group receiving SIM0235. Treatment commenced seven days post-inoculation, upon the formation of solid axillary tumours. Mice were administered the respective agents via intraperitoneal injection twice a week for a total of four doses. Tumour volumes and body weights were measured and recorded every three days throughout the study. Humane endpoints were applied, and mice were euthanized when the tumour volume exceeded 1500 mm³. Subsequently, tumours were excised and weighed. All animal procedures were approved by the Animal Ethics Committee of the Chinese Academy of Medical Sciences (Approval No. ACUC-XM5B-2025-163) and were conducted in strict compliance with institutional guidelines for the care and use of laboratory animals.

### Detection and qualification of actively translated ORFs

ORFs were detected in Ribo-seq data with ORFquant (v1.1.0) [18] and Ribotricer (v1.3.2) [19]. We required an ORF to be detected in at least 2/19 tumour samples or 2/15 paracancerous tissues. Moreover, the longest ORF was maintained if multiple ORFs had the same start or end coordinates. The final list of ORFs was generated by combining the filtered sets of ORFs in tumour and paracancerous tissues. The count of each ORF was determined by samtools (v1.3.1) [20] and normalized with DESeq2 (v1.30.1) [21].

Based on the Ribo-seq levels (transcripts per million (TPM) values), we defined an ORF as “tumour specific” when the average TPM value was in the top 90% across all tumour samples and in the bottom 10% across all paracancerous tissues. “Normal specific” and “both” ORFs were defined similarly. The group of “upregulated ORFs” comprises (a) the “tumour-specific” ORFs and (b) the subset of “both” ORFs that were significantly upregulated in tumours (*p* < 0.05 and log2FC > 0).

### Variant detection

Whole-exome PE150 sequencing was performed on an Illumina HiSeq platform with a 150 bp read length. Single-nucleotide variant (SNV)/indel calling and annotation were performed following the stepwise procedures of the Genome Analysis Toolkit (GATK) Best Practices pipeline [22]. In brief, read pairs filtered with Trimmomatic (v0.36) [23] were aligned to the human reference genome (hg38) by BWA-mem (v0.7.17) [24]. The mapped reads were further refined using Picard (v2.25.2) program suites and GATK (v4.2.0.0); the refinement process included removing PCR duplicate reads and realigning reads surrounding potential indel regions. A panel of controls was generated and used to call somatic variants with MuTect2 [25] in tumour-only mode. SNVs and indels were selected as high-confidence variants with GATK ‘FilterMutectCalls’ with a minimum read depth of 20, and ANNOVAR (v20200608) [26] was then used to perform variant functional annotation of the variants. The R package ‘maftools’ (v2.6.05) [27] was utilized to analyze the mutation profiles. Mutational signatures were analyzed using the R package ‘MutationalPatterns’ (v3.0.1) [28], which was also used to determine the contribution of “signatures of mutational processes in human cancer” (source http://cancer.sanger.ac.uk/cosmic/signatures*)* in CRC tissue and organoids.

### RNA-seq and Ribo-seq data analysis

To generate RNA-seq and Ribo-seq data, all samples were sequenced using the Illumina HiSeq X Ten or NovaSeq platform with a strand-specific paired-end 150-bp read length, and Read2 was used for subsequent data analyses. Adaptor sequences were trimmed with Cutadapt (v1.14) [29]. Reads with distinct inline barcodes were demultiplexed, and a 10-bp random sequence was appended to the read name using custom scripts. Low-quality reads were filtered with Trimmomatic (v0.36). For Ribo-seq data, only reads with lengths of 25–50 bp were retained. Prior to mapping, the reads were filtered for tRNAs, mitochondrial RNAs, and ribosomal RNAs using Bowtie2 (v2.2.9) [30]. Cleaned reads were mapped to the *Homo sapiens* genome (Ensembl GRCh38.p13) with STAR (v2.1.1) [31] using the default settings. Uniquely mapped reads retained by SAMtools (v0.1.19) [20] were then processed through a custom-built script to remove PCR duplicate reads based on shared identical random sequences. Next, the filtered .bam files were used to count the reads mapped to CDSs to quantify the gene expression level and translation abundance via ‘featureCounts’ (v1.6.3) [32]. DESeq2 (v1.30.1) [21] was used for differential gene expression analysis and gene count normalization. All genes with a nonzero count in the samples were considered. The TE of genes was calculated by taking the ratio of the Ribo-seq counts to the RNA-seq counts, and the deltaTE method in R [33] was used to identify genes that were differentially and translationally regulated. Genes with a *p* value < 0.05 were considered differentially expressed or differentially translated.

In addition, for the Ribo-seq data, 3-nt periodicity, P-site calibration and metagene read depth were analyzed with Plastid using the filtered .bam files [34]. For RNA-seq and Ribo-seq data, the lengths of RPFs and their distribution in the CDS, 5’ UTR and 3’ UTR of annotated protein-coding genes were determined with RiboProfiling (v1.22.0) [35].

### Weighted gene correlation network analysis (WCGNA)

All genes were filtered by the following criteria: (i) genes with a mean TE > = 1 across the 19 CRC samples and (ii) genes with a variation ranking in the top 70%. Then, the filtered genes were subjected to WGCNA to identify modules of highly correlated genes via the ‘WGCNA’ package in R software [36], with the following parameters: power = 9, mergecutHeight = 0.26, and minModuleSize = 35. The patients were stratified into two age subgroups: ≤60 and > 60 years. Subsequently, genes from the modules significantly (defined by *p* < 0.05 and R^2^ > 0) correlated with clinical traits or gene mutations of interest were selected for Gene Ontology (GO) and Kyoto Encyclopedia of Genes and Genomes (KEGG) pathway analyses. Hub genes were identified by the threshold criteria of |gene significance (GS) score| >0.4 and |module membership (MM) score| >0.7. The statistical significance of these module-trait associations was further corroborated using enrichment scores with the ‘GSVA’ package in R software [37].

### Enrichment analyses

GO and KEGG functional enrichment analyses were performed using the Metascape Database http://metascape.org/ [38]. Terms with *p* values < 0.05 were considered enriched.

### Comparative analysis of 5’UTR features

The genes exhibiting no significant changes in translational efficiency (*p* > 0.05) served as the control. For each feature —including uORFs, TOP elements, IRESs and m^6^A modification sites — the gene ratio was calculated as the proportion of genes within the feature. uORF analysis was based on the uORF list present in at least 2 of 19 samples detected by Ribotricer. The repertoire of TOP elements was curated from Sankaran VG’s Lab [39]. m^6^A sites list was retrieved from the database RMBase v3.0 [40] (https://rna.sysu.edu.cn/rmbase3/index.php), and a cumulative m^6^A motif score per gene was computed by summing the scores of all sites. IRES probability was predicted in silico using IRESpy [41].

### Statistics and reproducibility

All data (with error bars) are presented as the mean ± standard deviation (s.d.) of at least three independent experiments. Statistical significance for two-sample comparisons was determined using Student’s t-test (unpaired two-tailed). For all box plots, the median, 25th and 75th percentiles, and minimum and maximum values are shown. When asterisks are used to indicate statistical significance, * indicates a *p* value < 0.05, ** indicates a *p* value < 0.01, *** indicates a *p* value < 0.001 and **** indicates a *p* value < 0.0001. All the data were analysed using GraphPad Prism 7.0 software.

## Results

### Translational profiling guides the discovery of *de novo* ORFs in CRC

**Fig. 1:**
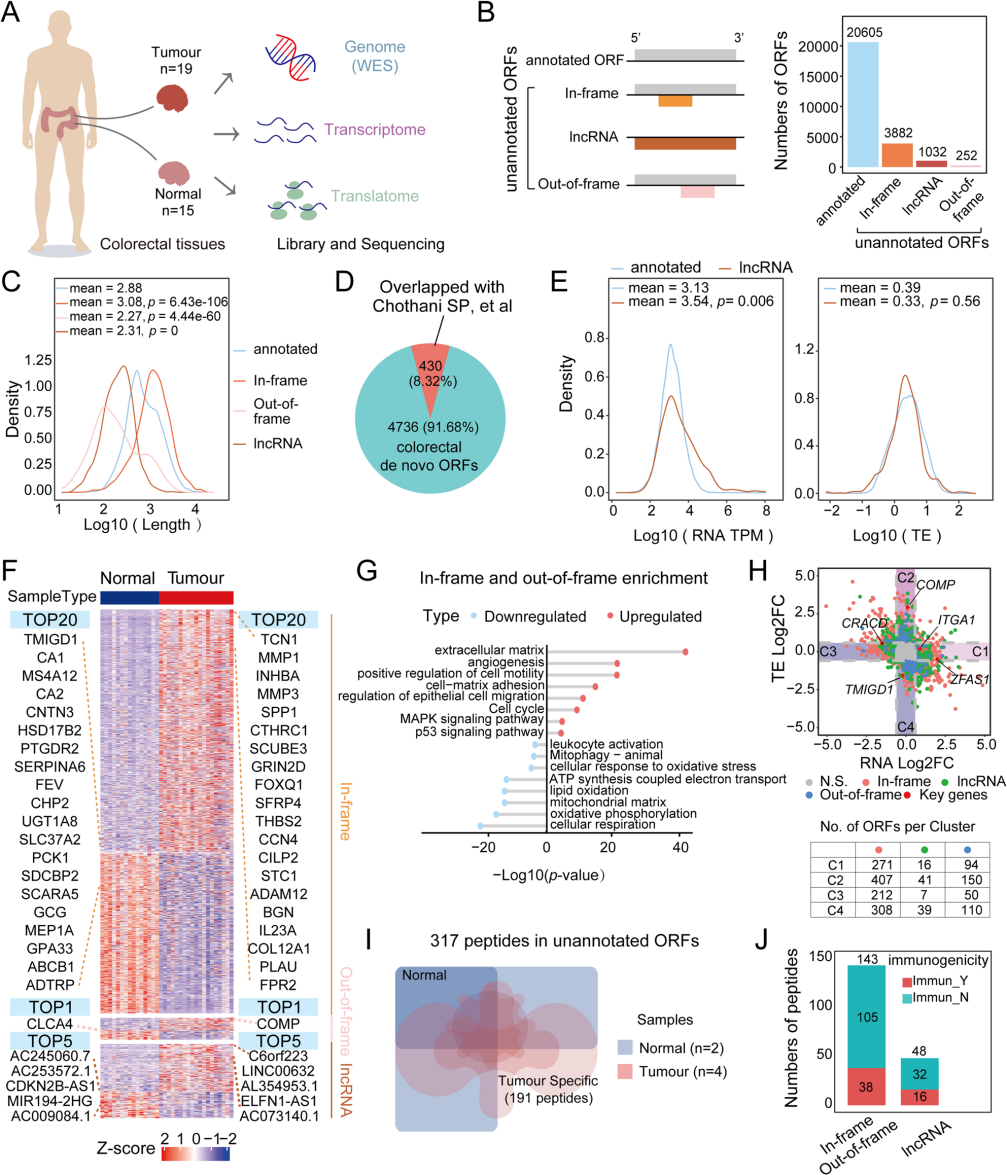
Identification of *de novo* ORFs and immunogenic peptides guided by translational profiles and the MHC-I immunopeptidome in CRC. **A** Schematic overview of the experimental workflow. **B** Summary of all identified ORFs. (Left) Schematic of the ORF classification strategy. (Right) Quantification of annotated versus unannotated ORF categories. Unannotated ORFs are further subdivided by their genomic origin. **C** Length distribution of each unannotated ORF category compared with annotated ORFs. **D** Pie chart showing the overlap between identified *de novo* ORFs and the sORFs reported by Chothani et al. **E** RNA-seq transcript per million (TPM; left) and translation efficiency (TE; right) distributions for lncRNA-derived ORFs and annotated ORFs. **F** Translational expression of *de novo* ORFs (Tumour: *n* = 19; Normal: *n* = 15). TOP genes (ranked by |log2FC|) are labeled for each group. The color scale applies to all panels. **G** Lollipop plot of representative GO terms and pathways enriched among genes with in-frame and out-of-frame ORFs (*p* < 0.05). **H** Scatter plot showing fold changes of unannotated ORFs at the transcriptional and translational levels. In-frame ORFs (light red), out-of-frame ORFs (blue), lncRNA-derived ORFs (green), and cancer-related genes (red) are highlighted. C1–C4 indicate the four expression-based ORF clusters. **I** Venn diagram showing the distribution of unannotated ORFs detected in four CRC tumour samples and two paracancerous tissues (Tumour: *n* = 4; Normal: *n* = 2). **J** Counts of the 191 tumour-specific peptides identified, categorized by ORF type. Statistical significance for two-sample comparisons was calculated using unpaired, two-tailed Student’s t-tests

To generate a high-resolution translatomic map, we collected 19 CRC tumour samples and 15 paired paracancerous tissues from 19 patients (Fig. S1A) for parallel mRNA sequencing (mRNA-seq) and ribosome profiling (Ribo-Seq; Fig. S1B), which allowed us to characterize the transcriptome and translatome of colorectal tissue, respectively (Fig. 1A). ORFs were identified through sequenced ribosomal footprints (also known as ribosome-protected fragments [RPFs]), which were predominantly ~ 30 nt in length (Fig. S1C). These ORFs mapped mainly to coding sequences (CDSs) (Fig. S1D) and displayed the expected 3-nt periodicity (Fig. S1E), consistent with previous studies [42].

Using ribotricer [19] and ORFquant [18], we identified a total of 25,771 ORFs, comprising 20,605 annotated ORFs and 5,166 *de novo* ORFs, also referred to as unannotated ORFs. Of the *de novo* ORFs, 4,134 originated from protein-coding genes (3,882 in-frame and 252 out-of-frame), and 1,032 lncORFs were derived from long noncoding RNAs (lncRNAs) (Fig. 1B and Fig. S1F-G; Table S3). Most lncRNA-derived and out-of-frame ORFs were shorter in length (Fig. 1C) and may serve as sources of micropeptides. Micropeptides are typically small functional proteins encoded by small open reading frames (sORFs), with lengths generally under 100 amino acids. They are widely involved in various life-sustaining activities across organisms, ranging from homeostasis regulation and disease processes to tumorigenesis, development, and morphogenesis in both animals and plants [43]. Our discovery paves the way for investigating the functions of these non-canonical translation products in CRC.

By contrast, the in-frame ORFs tended to be longer than their canonical counterparts (Fig. 1C), suggesting the use of alternative ribosomal reading frames via *de novo* start codons or read-through events. Notably, only 8.32% of these colorectal *de novo* ORFs overlapped with a previously reported dataset of small ORFs (sORFs) [44] from other human tissues (Fig. 1D), indicating that these noncanonical translational events are largely tissue-specific. We also observed slightly elevated RNA levels for lncORFs compared to annotated ORFs, while their translational efficiencies (TEs) were similar (Fig. 1E), suggesting that lncORFs preferentially arise from highly expressed lncRNA genes.

To investigate which *de novo* ORFs might be cancer-relevant, we next quantified and identified differentially expressed ORFs, especially those with expression changes in tumour tissues and the genes from which these ORFs originate (Fig. 1F). Interestingly, the upregulated in-frame and out-of-frame ORFs, both derived from protein-coding genes, were functionally enriched in tumorigenesis-related pathways, including angiogenesis, cell–matrix adhesion, and cell cycle regulation (Fig. 1G). Specifically, the most differentially expressed in-frame ORFs derived from the *TCN1* and *INHBA* genes—both implicated in CRC pathogenesis [45, 46]—may encode alternative protein isoforms with distinct functions. Moreover, we identified a tumour-specific lncORF from the *ELFN1-AS1* gene, which has been reported to regulate *MEIS1* expression by recruiting the EZH2-DNMT3A complex to the *MEIS1* promoter, contributing to CRC tumour growth and chemoresistance [47]. These findings suggest that lncRNAs such as *ELFN1-AS1* may function not only as noncoding RNAs but also as templates for micropeptide production in CRC.

We further classified these ORFs into four expression clusters (C1-C4) based on mRNA-seq and Ribo-seq data: C1 (381 ORFs, upregulated only at the transcript level), C2 (598 ORFs, upregulated only at the translational level), C3 (269 ORFs, downregulated only at the transcript level), and C4 (457 ORFs, downregulated only at the translational level) (Fig. 1H). Clusters C1 and C3 represent ORFs regulated exclusively at the transcriptional level, while C2 and C4 exhibit regulation at the translational level only, indicating that *de novo* ORFs, like canonical protein-coding genes, are subject to distinct regulatory modes.

Strikingly, many of these *de novo* ORFs originated from well-established CRC-associated genes, such as *ITGA1*,* COMP*,* and lncRNA ZFAS1*. *ITGA1* is recognized as a promising target for CRC diagnosis and treatment [48]; *COMP* can drive cell proliferation during early CRC tumorigenesis [49], and the *lncRNA ZFAS1* has been reported to promote CRC progression [50]. These findings raise the possibility that noncanonical translation expands the functional output of classical CRC driver genes.

### Translation of *de novo* ORFs expands the MHC class I immunopeptidome in CRC

Emerging evidence indicates that translation of noncoding genomic regions, including lncRNAs and untranslated regions (UTRs) of mRNAs, can give rise to noncanonical peptides that are actively involved in tumour immunity [51, 52]. To assess whether the *de novo* ORFs identified in CRC contribute functionally to tumour immunogenicity, we next examined their potential to generate peptides presented by the MHC class I complex in 4 of the 19 CRC tumour samples and 2 paired paracancerous tissues using mass spectrometry (MS).

The resulting immunopeptidome comprised 115,640 tumour-derived and 57,568 paracancerous tissue-derived peptides. Of these, approximately 90% were canonical peptides, and 38% to 71% of peptides in each sample were predicted to be immunogenic (see Materials and Methods, Fig. S1H, Table S4). In both tumour and paracancerous tissues, immunogenic MHC-I peptides were more frequently found as 9-mers than the nonimmunogenic peptides (Fig. S1I).

We also identified 3,638 peptides exclusively in tumour tissues, of which 1,631 were predicted to be immunogenic (Fig. S1J). These peptides may represent a rich source of tumour-specific antigens, substantially expanding the pool of potential therapeutic targets in CRC. Notably, we identified 317 peptides derived from *de novo* ORFs, 191 of which were CRC-specific (Fig. 1I). We further prioritized 54 peptides (38 from in-frame or out-of-frame ORFs and 16 from lncORFs) as potential candidates for tumour-specific neoantigens, based on their predicted immunogenicity (Fig. 1J).

Taken together, by using Ribo-seq to capture ribosomal footprints in CRC, we identified thousands of *de novo* ORFs, of which the tumour-specific *de novo* ORFs might provide a promising reservoir of CRC-specific neoantigens for immunotherapeutic development.

### Distinct contributions of transcriptional and translational dysregulation in CRC

**Fig. 2:**
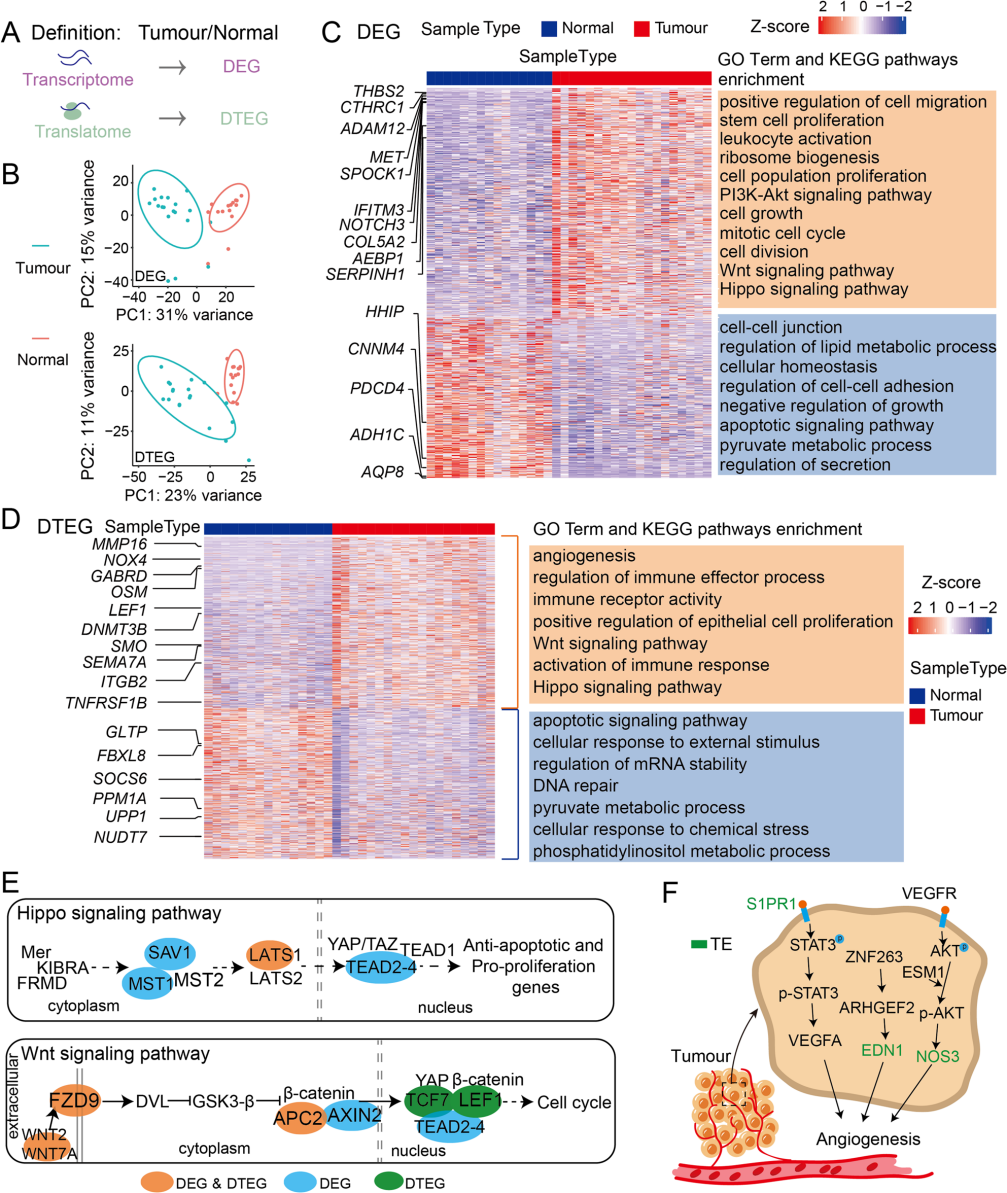
Regulation of coding sequence (CDS) ORFs, including DEGs and DTEGs, in CRC. **A** Diagram illustrating the definitions of differentially expressed genes (DEGs) and differential translation efficiency genes (DTEGs). **B** Principal component analysis (PCA) of tumour and paracancerous tissues based on transcriptome (upper panel) and translatome (lower panel) profiles. **C** Heatmap of DEGs identified by RNA-seq, showing representative genes (left) and enriched GO terms or pathways (right) (*p* < 0.05) (Tumour: *n* = 19; Normal: *n* = 15). **D** Heatmap of DTEGs with representative genes (left) and enriched GO terms or pathways (right) (*p* < 0.05) (Tumour: *n* = 19; Normal: *n* = 15). **E** KEGG pathway map showing transcriptional and translational regulation of genes in cancer-related pathways, including Wnt and Hippo signaling (*p* < 0.05). **F** Map for “angiogenesis” highlighting genes regulated specifically at the translational level

In addition to ORF identification, Ribo-seq enables the unbiased evaluation of translation efficiency (TE) globally (see Materials and Methods). By comparing CRC patients with controls, we defined differentially expressed genes (DEGs) as those with altered expression at the RNA level (transcriptome), and differential translation efficiency genes (DTEGs) as those with altered expression at the translational level (translatome) (Fig. 2A and Table S5). Both DEGs and DTEGs clearly distinguished tumour tissues from paracancerous tissues on principal component analysis (PCA) (Fig. 2B). Specifically, we identified 3,795 DEGs (2,237 upregulated and 1,558 downregulated) (Fig. 2C), which were enriched in biological processes such as cell migration, proliferation, and cancer-related signaling pathways, including the Wnt and Hippo pathways. Of note, the DEGs identified in our RNA-seq dataset showed a substantial overlap with those from the TCGA CRC cohort (Fig. S2A). For instance, *MET*, *IFITM3*, *NOTCH3*, and *COL5A2* were upregulated in CRC, while *HHIP* and *PDCD* were downregulated. In parallel, we detected 1,257 DTEGs (668 upregulated and 589 downregulated) (Fig. 2D), which are enriched in pathways related to angiogenesis, immune responses, and DNA repair (Fig. 2D and Fig. S2B).

The distinct functional profiles of DEGs and DTEGs highlight the importance of translatome analysis in uncovering novel molecular mechanisms in tumorigenesis. Interestingly, both DEGs and DTEGs included genes from the Wnt and Hippo signaling pathways (Fig. 2C-D), suggesting coordinated transcriptional and translational regulation. For instance, *SAV1*, *MST1*, and *TEAD2–4* from the Hippo pathway, and *AXIN2* from the Wnt pathway, were primarily regulated at the transcriptional level. In contrast, key components of Wnt signaling such as *WNT2*, *WNT7A*, *FZD*, and *APC2*, and *LATS1* showed coordinated regulation at both transcriptional and translational levels. Notably, *LEF1* and *TCF7*, also part of the Wnt pathway, were regulated exclusively at the translational level (Fig. 2E). Strikingly, the “angiogenesis” pathway seemed to be restricted towards translational regulation, as the expression of such genes, including *S1PR1*, *EDN1*, *NOS3*, was regulated only at the translational level (Fig. 2F).

Together, these findings reveal previously unappreciated translational regulation in pathways central to CRC development.

### Translationally regulated gene modules correlate with clinical traits and genetic alterations in CRC

The role of translational regulation in CRC oncology remains largely unclear, particularly in relation to the genomic and clinical heterogeneity of CRC patients. To address this, we first characterized the somatic mutation landscape of the recruited CRC cohort. 18 of the 20 most frequently reported CRC alterations [3, 53] were present in the majority (16 out of 19) of our CRC patient samples (Fig. S3A and Table S6). Moreover, the mutation frequencies closely mirrored those reported in the TCGA CRC dataset (Fig. S3A; see Materials and Methods).

**Fig. 3:**
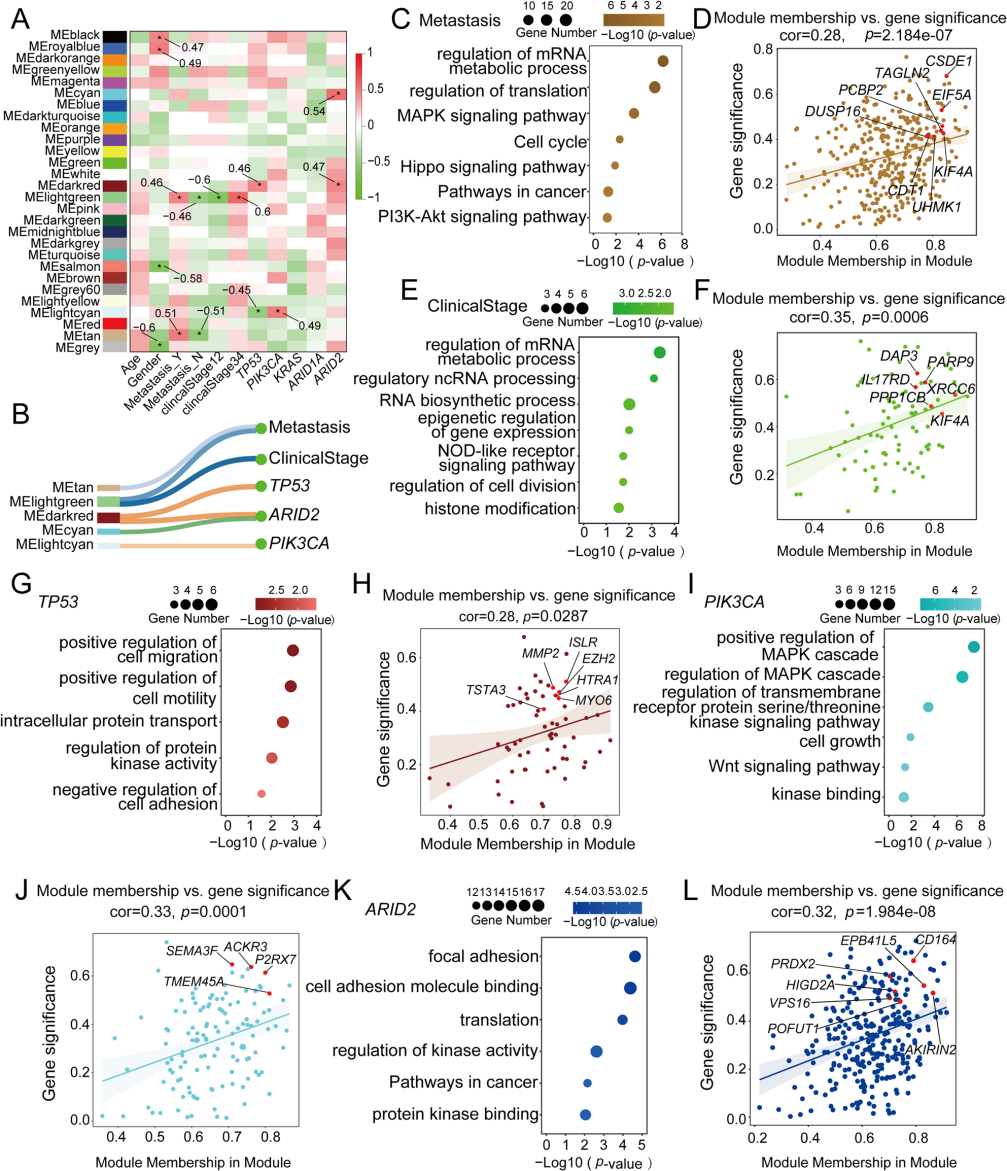
Clinical relevance of translational dysregulation in CRC. **A** Heatmap showing associations between translatomic modules and clinical traits or gene mutations (* indicates significant associations). **B** Diagram highlighting five modules significantly associated with major clinical features (metastasis and clinical stage) and representative mutations (TP53, PIK3CA, and ARID2). **C**,** D** GO enrichment plot (**C**, *p* < 0.05) and interconnectivity plot (**D**) showing functional terms and hub genes in modules associated with metastasis. **E**,** F** GO enrichment plot (**E**, *p* < 0.05) and interconnectivity plot (**F**) for the module associated with clinical stage. **G**,** H** GO enrichment plot (**G**, *p* < 0.05) and interconnectivity plot (**H**) for the module associated with TP53 mutation. **I**,** J** GO enrichment plot (**I**, *p* < 0.05) and interconnectivity plot (**J**) for the module associated with PIK3CA mutation. **K**,** L** GO enrichment plot (**K**, *p* < 0.05) and interconnectivity plot (**L**) for the module associated with ARID2 mutation. Key cancer-related genes (|MM score| > 0.7, |GS score| > 0.4) are shown in red

We then performed weighted gene correlation network analysis (WGCNA; see Materials and Methods) based on translatome expression data, identifying 28 distinct gene “modules” (Fig. 3A). These modules were correlated with clinical traits (age, sex, metastasis status, and disease stage) and frequently mutated genes in CRC (*TP53*, *PIK3CA*, *KRAS*, *ARID1A*, and *ARID2*) (Fig. 3A). We identified modules that significantly correlated (*p* < 0.05) with metastasis (MEtan and MElightgreen), advanced clinical stage (MElightgreen), and mutations in *TP53* (MEdarkred), *PIK3CA* (MElightcyan), and *ARID2* (MEdarkred and MEcyan) (Fig. 3B). The majority of all module-trait associations were further corroborated by Gene Set Variation Analysis (GSVA) (Fig. S3B-C).

Specifically, MElightgreen and MEtan, both associated with metastasis—the leading cause of CRC-related mortality [54]—were enriched for genes undergoing active translation in MAPK and PI3K/Akt signaling pathways (Fig. 3C), suggesting that translational control plays a specific role in CRC metastasis. Based on gene significance (GS) and module membership (MM) scores, we identified 341 hub genes from these modules, including *CSDE1*, *PCBP2*, *KIF4A* and *EIF5A* (Fig. 3D). Notably, high mRNA levels of *CSDE1* and *PCBP2* have been previously associated with poor prognosis in CRC [55–57].

Modules correlated with advanced CRC stage (III/IV) were functionally enriched in RNA processing and epigenetic regulation (Fig. 3E) and included 89 hub genes such as *XRCC6*, *KIF4A*, *PPP1CB* and *PARP9* (Fig. 3F). Among these, upregulation of *DAP3* has been shown to accelerate tumour progression across multiple cancer types, including CRC [58–60].

We also explored the relationship between translatome changes and common gene mutations. Mutations in *TP53*—present in ~ 50% of CRC cases—are linked to lymphatic invasion and poor survival [61]. The TP53-associated module was enriched for genes involved in cell motility and adhesion, suggesting a role in promoting migration and invasion (Fig. 3G). The 62 hub genes in this module included *ISLR*, *EZH2* and *MYO6* (Fig. 3H), all previously implicated in CRC invasion or metastasis [62–67].

Similarly, clinical mutations in *PIK3CA* are strongly associated with poor outcomes [68]. The associated module was enriched for genes involved in the MAPK cascade, cell growth, and transmembrane receptor serine/threonine kinase signaling (Fig. 3I). We also observed links between *PIK3CA* mutations and the Wnt signaling pathway, suggesting crosstalk between PI3K, MAPK, and Wnt pathways in CRC tumorigenesis [69]. This module contained 130 hub genes, including *TMEM45A*, *P2RX7*, *ACKR3*, and *SEMA3F* (Fig. 3J). Notably, the upregulation of *SEMA3F* has been shown to drive tumorigenesis in hepatocellular carcinoma (HCC) patients [70], highlighting a possible shared mechanism across cancers.

Finally, *ARID2* mutations—also frequent in CRC [71]—are associated with shorter progression-free survival [72]. The mutations were linked to two modules enriched in cell adhesion and kinase activity (Fig. 3K). Among the 299 hub genes in these modules were *POFUT1*, *PRDX2*, *EPB41L5*, *HIGD2A*, *AKIRIN2*, *CD164*, and *VPS16* (Fig. 3L), all of which regulate key pathological processes in various cancers [73–79]. For example, *POFUT1* promotes CRC development [73], *PRDX2* is associated with tumorigenesis and metastasis in CRC [74, 80], and *EPB41L5* mediates TGFβ-driven metastasis in gastric cancer [75].

Together, these findings support a novel translation-dependent regulatory mechanism that contributes to CRC progression and links translational control with specific gene mutations and clinical features.

### Differentially transcriptional and translational control of gene expression in CRC

**Fig. 4:**
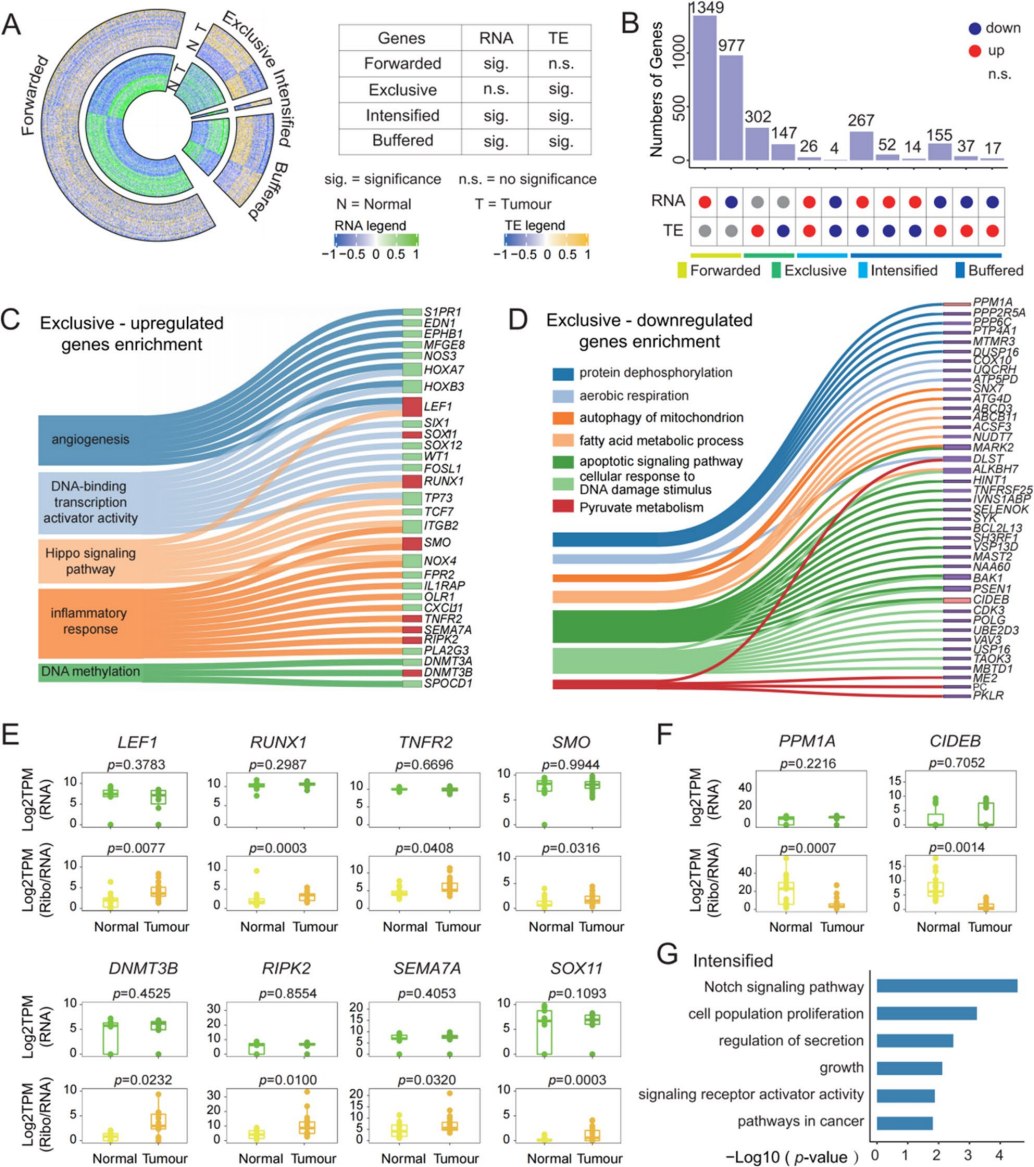
Differential gene expression specifically associated with CRC. **A** Circular heatmap showing four distinct classes of DEGs and DTEGs (Tumour: *n* = 19; Normal: *n* = 15). **B** Bar plot displaying the number of genes in each class. **C** Sankey diagram of representative GO terms and pathways enriched in the 302 upregulated DTEGs in the Exclusive class (*p* < 0.05); key cancer-related genes are highlighted in red. **D** Sankey diagram of representative GO terms and pathways enriched in the 147 downregulated DTEGs in the Exclusive class (*p* < 0.05). **E** Box plots showing expression changes of representative upregulated DTEGs in the Exclusive class. **F** Box plot showing expression changes of representative downregulated DTEGs in the Exclusive class. **G** GO enrichment terms and pathways (*p* < 0.05) for genes in the Intensified class

Given the critical role of translational regulation in CRC, we categorized all gene hits identified as DEGs and DTEGs into four distinct classes: (i) *Forwarded* — 2,326 genes regulated exclusively at the transcriptional level; (ii) *Exclusive* — 449 genes regulated exclusively at the translational level; (iii) *Intensified* — 30 genes coordinately regulated at both levels; and (iv) *Buffered* — 542 genes whose expression was shaped by opposing transcriptional and translational regulation (Fig. 4A-B).

We first focused on the *Exclusive* class (Fig. S4A), as these genes are predominantly subject to translational control. This group included 302 upregulated genes enriched in pathways such as angiogenesis, inflammatory responses, Hippo signaling, and DNA methylation (Fig. 4C), as well as 147 downregulated genes involved in aerobic respiration, DNA damage response, and metabolic processes including fatty acid and pyruvate metabolism (Fig. 4D). Dysregulation of mitochondrial homeostasis [81] and DNA repair [82] has been linked to CRC cell growth, and our findings suggest these processes may be governed in part by translational mechanisms.

Among the translationally upregulated *Exclusive* genes, we identified eight well-characterized cancer-associated regulators: *LEF1*, *DNMT3B*, *RUNX1*, *RIPK2*, *TNFR2*, *SEMA7A*, *SMO* and *SOX11* (Fig. 4E). *LEF1* is a component of the Wnt/β-catenin signaling axis and activates c-Myc and cyclin D1 in CRC [83]. *DNMT3B*, a DNA methyltransferase, is frequently overexpressed in CRC and linked to increased tumour invasiveness, metastasis [84], and poor clinical outcomes [85]. *RIPK2*, a master regulator of immune and inflammatory response, shows markedly elevated expression in colorectal tumours relative to paracancerous tissues. Its role in CRC progression is supported by its modulation of BIRC3-mediated ubiquitination of IKBKG, and activation of oncogenic NF-κB signaling [86, 87]. *TNFR2*, a cell surface receptor involved in cell survival and proliferation, is predominantly expressed in immunosuppressive cells, such as Tregs [88, 89], and its soluble form (sTNFR2) is associated with increased CRC risk [88]. Intriguingly, we found these genes exhibit translation-dominant rather than transcription-dominant regulation, revealing an unconventional mode of expression control.

Conversely, translationally downregulated genes in the *Exclusive* class included *PPM1A*—a Mg²⁺/Mn²⁺-dependent phosphatase that inhibits the TGF-β signaling pathway and whose loss is linked to poor overall survival in CRC patients—and the less well-characterized apoptosis regulator *CIDEB *[90] (Fig. 4F). These patterns further support the presence of distinct post-transcriptional regulatory networks shaping CRC pathogenesis.

We also identified 30 *Intensified* genes, which exhibited synergistic regulation at both transcriptional and translational levels. These genes were enriched in the Notch signaling pathway and cell proliferation processes (Fig. 4G and Fig. S4B). Notable examples include *STRA6* and *NOTCH4*. *STRA6* encodes a multi-domain membrane protein that serves as the cell surface receptor for retinol-binding protein (RBP) involved in vitamin A homeostasis [91]; *NOTCH4* serves as a key component of Notch signaling [92]. Our data suggest that such tumour-specific alterations in these genes may play critical roles in CRC, as their regulation is under intensified control at both transcriptional and translational levels.

### TNFR2 is selectively upregulated through translational control in CRC

**Fig. 5:**
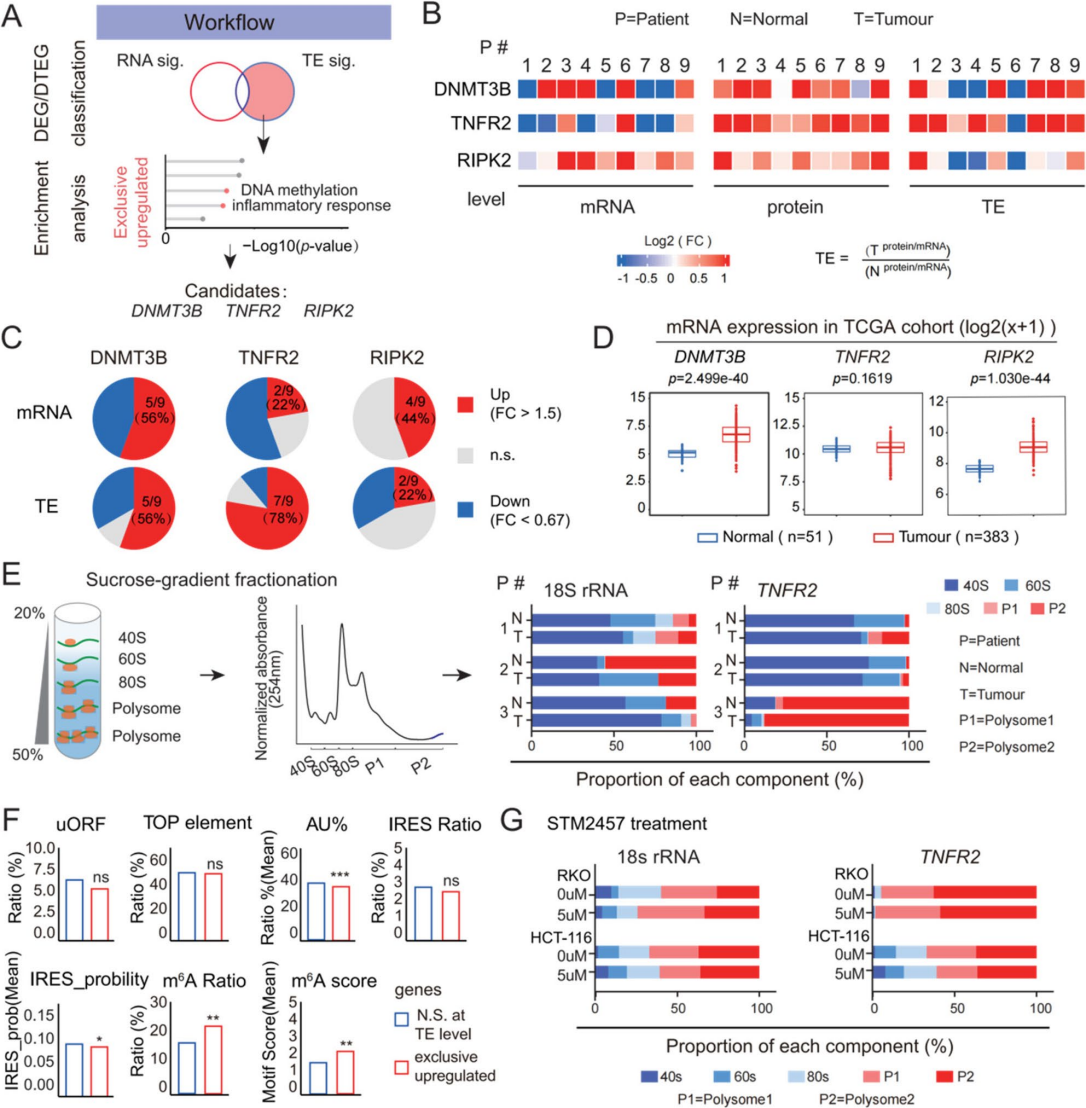
Multilevel expression analysis of candidate genes and upstream regulatory mechanism of TNFR2 in CRC. **A** Workflow for identifying candidate genes regulated at the translational level. **B** Fold changes in expression at the transcriptional, translational efficiency (TE), and protein levels for each gene across the nine pairs. **C** Proportion of samples exhibiting significant dysregulation of each gene among the nine paired samples. **D** Box plot showing *DNMT3B*, *TNFR2*, and *RIPK2* mRNA expression in the TCGA CRC cohort (Tumour: *n* = 383; Normal: *n* = 51). **E** Left: polysome profiling of tumour and paracancerous tissues. Right: qPCR analysis of *TNFR2* mRNA in polysome fractions; 18 S rRNA was used as an internal control (Normal, Tumour; *n* = 3 pairs). **F** Bar plot displaying the Ratio of genes with uORF, TOP element, the mean AU content in the 5’UTR, the Ratio of genes with predicted IRESs, the mean prob_IRES by IRESpy, the Ratio of genes with m^6^A site, and the meat cumulation motif score. For gene ratio comparisons, statistical significance was determined by Fisher’s exact test. For comparisons of continuous metrics, the two-sided Wilcoxon rank-sum test was used. **G** qPCR analysis of *TNFR2* mRNA in polysome fractions after SIM2457 (5 µM, 48 h) or DMSO (vehicle) treatment; 18 S rRNA was used as an internal control

Since the *Exclusive* class includes genes regulated solely at the translational level, we specifically investigated this group to better understand the pathological role of translational dysregulation in CRC (Fig. 5A). Among the translationally upregulated genes in *Exclusive* class, *DNMT3B*, *RIPK2*, and *TNFR2* stood out due to their known roles in cancer-related processes [93–96] and strong TE shifts.

We validated the expression patterns of these three candidates at the mRNA, protein, and translational levels in nine paired CRC and matched paracancerous tissue samples. The mRNA expression levels varied: *DNMT3B* showed inconsistent changes across samples, *TNFR2* tended to be downregulated in CRC compared to controls, while *RIPK2* was generally upregulated (Fig. S5A). By contrast, their protein levels were consistently elevated in CRC tissues (Fig. S5B). Further calculation of TE revealed TNFR2 with the most pronounced translational upregulation (Fig. 5B), consistent with our sequencing data (Fig. 5C). Notably, in the TCGA CRC cohort, *TNFR2* mRNA levels showed no significant difference between tumour and paracancerous tissues (Fig. 5D), suggesting that its role may have been overlooked in transcriptome-based analyses.

To further validate translational regulation of *TNFR2*, we performed polysome profiling followed by quantitative PCR (qPCR) in CRC tumour and paracancerous tissues. Compared with paracancerous tissues, *TNFR2* mRNA in patient tissues exhibited a significant increase in the polysome fractions (Fig. 5E). These findings demonstrate that *TNFR2* upregulation in CRC is primarily driven by translational rather than transcriptional mechanisms. To investigate the translational regulatory mechanism, we assessed the major features within the 5’ untranslated region (5’UTR) known to mediate translation efficiency, including upstream open reading frames (uORFs), TOP elements, AU-rich elements (AREs), internal ribosome entry sites (IRESs), and N6-methyladenosine (m^6^A) modification sites. Comparative analysis revealed that m^6^A was relatively enriched in the translationally upregulated *Exclusive* genes, including *TNFR2* (Fig. 5F). Previous studies have indicated that m^6^A activation promotes key pathological features of CRC, including tumour progression, hepatic metastasis, and resistance to chemotherapy. Accordingly, such components of the m^6^A regulatory machinery—including *METTL3*, *RBM15*, *ZC3H13* and *YTHDF1*—showed increased translation in CRC compared with normal controls (Fig. S5C), supporting the activation of an m^6^A axis in tumours. To test this hypothesis, we employed the METTL3 inhibitor STM2457 in CRC cell lines to reduce m^6^A methylation levels (Fig. S5D). Pharmacological inhibition of METTL3 decreased the translation efficiency of *TNFR2*. Specifically, in RKO cells, the proportion of *TNFR2* mRNA in polysome 2 fraction declined upon inhibitor treatment, while in HCT116 cells, it decreased in both polysome 1 and 2 fractions (Fig. 5G, Fig. S5E). Collectively, these data indicate that the translational upregulation of *TNFR2* might be through an m^6^A-dependent program.

### TNFR2, which is translationally activated, promotes CRC progression

**Fig. 6:**
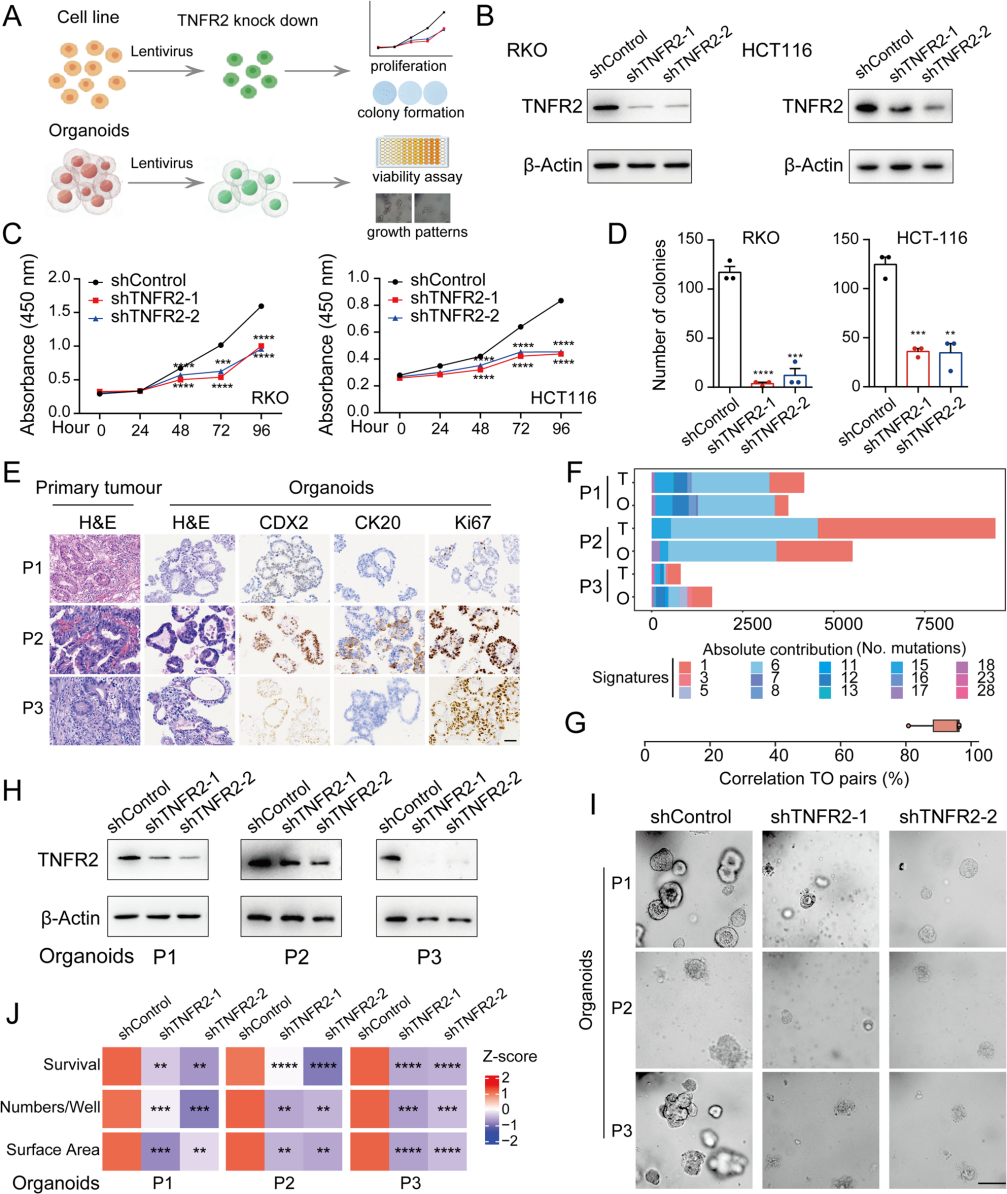
Targeting TNFR2 inhibits colorectal cancer progression. **A** Schematic overview of the experimental strategy for functional validation of TNFR2. **B** Western blot analysis of TNFR2 protein expression in RKO and HCT116 cells upon TNFR2 knockdown. **C** Proliferation assay results for TNFR2-knockdown RKO and HCT116 cells (*n* = 3). **D** Quantification of colony numbers in colony formation assays following TNFR2 knockdown (*n* = 3). **E** Comparative histological and immunohistochemical staining of CRC tissues and matched patient-derived organoid lines. **F** Stacked bar graphs showing total mutation burden per mutational signature in each patient tissue-organoid pair (T: tissue; O: organoid). **G** Box-and-whisker plot showing correlations (percentage) of mutational spectra between tissue and organoid pairs (T: tissue; O: organoid; *n* = 3 pairs). **H** Western blot analysis of TNFR2 protein expression in CRC organoids with TNFR2 knockdown (*n* = 3). **I** Morphological changes observed in organoids after TNFR2 knockdown (*n* = 3). Scale bar: 100 μm. **J** Quantification of changes in organoid viability, number, and surface area following TNFR2 knockdown (*n* = 3)

While TNFR2 is known for its immunoregulatory role in Tregs [96, 97], its function within tumour cells remains poorly defined. Since our data suggested TNFR2 is significantly upregulated at translational levels, we propose that TNFR2 may act as a tumour-intrinsic oncogenic factor in CRC, independent of its immune functions. To test this hypothesis, we used CRC cell lines (RKO and HCT116) and patient-derived organoid models to assess the role of *TNFR2* in CRC tumorigenesis (Fig. 6A). Genetic depletion of *TNFR2* (Fig. 6B, Fig. S6A) significantly impaired proliferation in both cell lines (Fig. 6C) and reduced their clonogenic capacity (Fig. 6D, Fig. S6B), indicating that TNFR2 supports CRC cell growth.

To further validate these results in a clinical-relevant 3D model, we established patient-derived CRC organoids (Fig. S6C). Histological and immunohistochemical analyses confirmed that the organoids retained key features of the original tumour, including expression of CDX2, CK20, and Ki67 (Fig. 6E). To validate the relevance of our organoid model, we performed whole exome sequencing (WES) on matched tumour tissues and organoids (Table S7). Although mutation burden varied across patients (e.g., higher in P1 and P2 than in P3), overall genomic concordance between organoids and their parental tumours was high, with > 80% mutation spectrum similarity (Fig. 6F–G and Fig. S6D–E). Organoids also retained key CRC driver mutations, including *KRAS*,* BRAF*, and *PIK3CA* (Fig. S6F), supporting their use for functional TNFR2 studies. Upon *TNFR2* knockdown (Fig. 6H, Fig. S6G), CRC organoids exhibited progressive structural disintegration, including complete loss of self-organization and intercellular cohesion (Fig. 6I). Correspondingly, their proliferation and growth were significantly impaired, and organoid size was markedly reduced (Fig. 6J). These findings confirm that TNFR2 upregulation is required to maintain CRC tumour architecture and growth.

### Pharmacological inhibition of TNFR2 suppresses CRC tumorigenesis

**Fig. 7:**
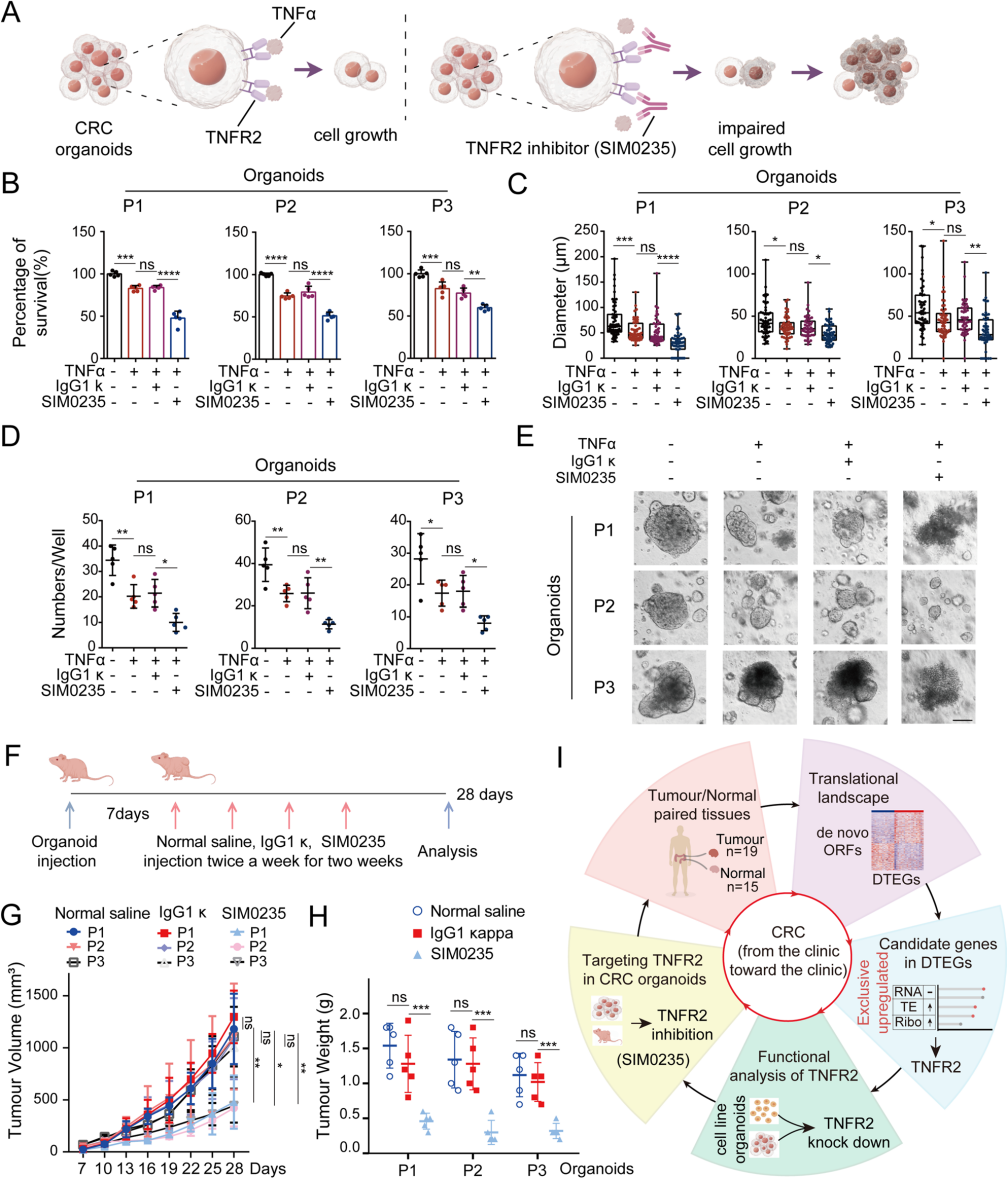
Targeting TNFR2 with inhibitors suppresses colorectal cancer progression. **A** Schematic overview of the experimental strategy for TNFR2 inhibitor validation (By Figdraw). **B** Survival rates of organoids from three patients after 72 h of SIM0235 treatment (*n* = 5). **C** Diameter measurements of organoids (*n* = 50) after 72 h of drug treatment. **D** Organoid counts (diameter > 25 μm) per well following 72 h of SIM0235 treatment (*n* = 5). **E** Representative images showing organoid morphology after 72 h of treatment. Scale bar: 50 μm. **F** Schematic of the in vivo therapeutic protocol (By Figdraw), the arrows indicate the time points of treatment intervention. **G**,** H** Tumour volume (**G**) and tumour weight (**H**) in mice treated with drugs (*n* = 5 per group). **I** Graphical summary illustrating how translational profiling reveals novel insights into CRC carcinogenesis

TNFR2 acts as the transmembrane receptor of TNFα [98]. Finally, to investigate whether TNFR2 represents a therapeutic vulnerability in CRC, we performed proof-of-concept studies using SIM0235 (Fig. 7A). SIM0235 is a clinical-stage TNFR2-specific inhibitor currently in phase I clinical trials for advanced solid tumours and cutaneous T-cell lymphoma (CTCL) (NCT05569057). SIM0235 treatment reduced organoid survival (Fig. 7B), confirming the oncogenic role of TNFα/TNFR2 in CRC. Notably, in the presence of TNFα, organoids treated with SIM0235 exhibited significant reduction in size (Fig. 7C), number (Fig. 7D), and structural integrity (Fig. 7E), suggesting that SIM0235 effectively suppresses CRC organoid growth by targeting TNFR2.

To evaluate in vivo efficacy, we established patient-derived organoid xenograft (PDOX) models using BALB/c-nu mice. This strain lacks T cells but retains functional monocytes/macrophages capable of endogenous TNFα production. Therefore, it is not necessary for exogenous TNFα administration. Mice were administered SIM0235 twice weekly for two weeks (Fig. 7F). This SIM0235 dosage significantly reduced tumour growth (Fig. 7G-H and Fig. S7A) without affecting body weight (Fig. S7B), demonstrating both high efficacy and tolerability. These results strongly support the clinical translational potential of TNFR2 inhibition as a therapeutic strategy for CRC.

In summary, our findings further underscore the importance of translational regulation in CRC and highlight TNFR2 as a promising therapeutic target. Inhibition of TNFR2—whose expression is specifically upregulated at translational level in CRC—represents a novel and effective strategy for limiting tumour progression (Fig. 7I).

## Discussion

Translational regulation has been shown to influence tumour initiation, progression, metastasis, and drug resistance across various cancers, including CRC [99–101]. A deeper understanding of translational control could enable the identification of more effective therapeutic targets and improve patient outcomes [102–105]. For example, Wiegering et al. reported that MYC-dependent proliferation in CRC cells can be suppressed by targeting the translation initiation factor eIF4A, thereby inhibiting MYC translation [106]. These findings underscore the urgent need for a comprehensive understanding of translational regulation in CRC.

In this study, we comprehensively characterized the translational landscape of CRC, uncovering thousands of novel ORFs, including several that are tumour specific. While noncanonical ORFs have often been overlooked in previous studies, growing evidence suggests they can be translated into functional peptides that contribute to tumour initiation and may serve as immunotherapy targets [107]. By integrating immunopeptidome and Ribo-seq analyses, we identified 191 immunogenic peptides derived from CRC-specific unannotated ORFs. These peptides—identified via MS and Ribo-seq—significantly expand the known repertoire of CRC neoantigens and may inform future immunotherapy strategies.

Our results further revealed that the change of unannotated ORFs in CRC is not only driven by transcriptional regulation but also governed by translational control. This highlights a critical yet underexplored mechanism in CRC biology, particularly in the production of noncanonical peptides. On the basis of the comprehensive depiction of the CRC translatome landscape provided above, we investigated translational regulation of canonical protein-coding genes and uncovered key dysregulation events. *TNFR2* emerged as a prominent example of translationally upregulated expression. Preliminary evidence suggests this upregulation correlates with m^6^A modification.

### Fundamental research implications

Our study provides the first translatome landscape of CRC, revealing widespread translational reprogramming events. We demonstrate that immune-related genes like *TNFR2* are translationally upregulated in CRC, adding a crucial regulatory dimension to our understanding of oncogene expression. The discovery of numerous novel tumour-specific ORFs and translationally controlled events offers a valuable foundational resource for the field, facilitating future exploration such as biomarker discovery and expanding the functional annotation of the cancer genome.

### Clinical research implications

Our findings hold significant translational potential. The identified immunogenic peptides from unannotated ORFs constitute a novel pool of potential targets for personalized immunotherapy, including neoantigen vaccines or T-cell therapies. More directly, we elucidate that *TNFR2* expression in CRC tumour cells is driven by translational upregulation. Using the TNFR2-targeting antibody SIM0235, we demonstrated significant growth inhibition of CRC both in vivo and in vitro, revealing a direct cytotoxic effect of TNFR2 blockade on tumour cells themselves. Currently, only one dual TNFR1*/*TNFR2 agent—tasonermin—is approved for clinical use, primarily for sarcoma. Several TNFR2-targeting monoclonal antibodies, including BI-1808 and SIM0235, are undergoing clinical evaluation. However, the focus of these agents has largely been on the cytotoxic effect of TNFR2^+^ Treg cells. Thus, this finding extends the therapeutic rationale for targeting TNFR2 beyond its established role in Treg-mediated immune suppression [97]. It opens the possibility for dual-action strategies simultaneously targeting TNFR2 in both immune and tumour compartments, or for combining translation inhibitors with TNFR2-directed therapies.

### Limitations and future directions

This study has limitations. The sample size, while sufficient for robust landscape analysis, may not capture the full heterogeneity of CRC. These findings warrant further validation in larger, independent cohorts. Additionally, our study identified several potential immunogenic peptides. The lack of functional validation of these putative immunogenic peptides may limit their clinical translational relevance. Furthermore, a limitation inherent to the PDOX platform used in vivo validation is the absence of a mature adaptive immune system, which precludes evaluation of SIM0235’s impact on regulatory T cells (Tregs). Given the high expression of TNFR2 on Tregs and its context-dependent immunomodulatory roles, future studies in immunocompetent models and human organoid-T cell co-culture systems are warranted to elucidate how TNFR2 blockade synergistically modulates both tumour cells and the immune microenvironment.

In conclusion, our findings underscore the clinical potential of targeting translationally regulated pathways like TNFR2 in CRC and highlight a broader opportunity for translation-focused cancer therapeutics. However, key challenges remain. While translational dysregulation is a cancer hallmark, current translation inhibitors lack sufficient specificity. Thus, the systematic identification of tumour-specific translational vulnerabilities, as initiated here, is of great fundamental and clinical importance for enabling precision targeting. Overcoming these hurdles could substantially improve cancer treatment by merging the specificity of targeted therapy with the broad applicability of translational control.

## Conclusions

Our study establishes a systematic framework bridging unbiased translatome discovery to functional validation. We first performed an integrated multi-omics analysis of CRC, with a focus on the translatome, to provide a panoramic view of CRC translational atlas. This revealed a previously unrecognized, translationally driven oncogenic program encompassing novel open reading frames and possible immunopeptides. From this resource, we identified *TNFR2* as a key translation-dependent vulnerability with high clinical potential. We then functionally validated TNFR2 as a potential therapeutic target, demonstrating its efficacy in suppressing tumour progression. This work offers a compelling proof-of-concept for targeting translation-dependent pathways in CRC.

## Supplementary Information

Supplementary Material 1.

Supplementary Material 2. Supplementary Figure S1. Basic information on active translation and the MHC-I immunopeptidome in CRC (related to Figure 1). A Clinical information of 19 CRC patients for omics analysis. B Dot plot showing the proportion of raw sequence reads mapped to tRNAs, mitochondrial genome (MT), ribosomal RNAs (rRNAs), and canonical genes (“clean”) in human CRC Ribo-seq data. Only cleaned reads were used for further analysis. C Beeswarm plot showing the distribution of ribosome footprint lengths across all 34 samples. D Bar plot showing the percentage of reads mapping to the coding sequence (CDS) and 5'/3' untranslated regions (UTRs) of annotated protein-coding genes. Each line represents one sample. E Bar plot showing P-site positions derived from ribosome footprints across the first 150 nt of annotated ORFs (left), and the percentage of footprints aligning with the primary reading frame (right). F Histograms showing gene expression levels measured by Ribo-seq across all 34 samples. Genes passing the 1 TPM cutoff and identified as actively translated by ribotricer are highlighted in red. G Bar plot showing the number of ORFs identified by ribotricer in each sample. H Summary statistics of peptides in six samples. Peptides are classified as immunogenic (Immun_Y, dark) or non-immunogenic (Immun_N, light), and as canonical (C) or noncanonical (NC). I Left: length distribution of peptides across samples. Right: length distribution of immunogenic vs. non-immunogenic peptides. J Heatmap showing peptide profiles across six samples, including peptide counts for each class. Supplementary Figure S2. Regulation of CDS ORFs in the CRC Ribo-seq dataset (related to Figure 2). A Heatmap showing differentially expressed genes (DEGs) in the TCGA CRC cohort (left) and in the internal CRC cohort (right) (Tumour: n=19; Normal: n=15). B GO plot showing representative Gene Ontology (GO) terms and pathways enriched among DEGs and DTEGs (p < 0.05). Supplementary Figure S3. Somatic mutational profile of 19 CRC patients and GSVA-based validation of WGCNA module-trait associations (related to Figure 3). A Oncoplot displaying the most commonly mutated genes in tumour tissues from 19 CRC patients. Samples are ordered by total mutation count. Mutation frequencies in this cohort are compared with those in the TCGA CRC cohort (right panel). B Heatmap showing the correlations between WGCNA-identified modules and their corresponding clinical traits or gene mutations, as re-evaluated by GSVA. C Box plots showing the GSVA enrichment scores for each significant WGCNA-identified module across sample groups (e.g., metastasis: Y vs N). Supplementary Figure S4. Differential gene expression specifically associated with CRC (related to Figure 4). A Heatmap showing DTEGs in the Exclusive class (Tumour: n=19; Normal: n=15). B Box plots showing expression changes of representative genes in the Intensified class. Supplementary Figure S5. Expression analysis of candidate genes and m6A-related genes in tissues (related to Figure 5). A Relative mRNA expression levels of DNMT3B, TNFR2, and RIPK2 in nine paired tumour and paracancerous tissue samples (Normal, Tumour; n=9 pairs). B Western blot analysis of DNMT3B, TNFR2, and RIPK2 protein expression in the same paired tissue samples. C Heatmap showing the expression of m6A-related genes at the transcript and translational levels (Tumour: n=19; Normal: n=15). D Detection of m6A content per 200 ng of total RNA in RKO and HCT116 cell lines after SIM2457 (5 µM, 48 hours) or DMSO (vehicle) treatment. E Polysome qPCR analysis of TNFR2 expression in RKO and HCT116 cells after SIM2457 (5 µM, 48 hours) or DMSO (vehicle) treatment; 18S rRNA was used as an internal control. Supplementary Figure S6. Tumour-derived organoids faithfully retain the histological architecture and genomic characteristics of the primary tissues (related to Figure 6). A Quantification of TNFR2 protein expression (normalized to β-Actin) in RKO and HCT116 cells upon TNFR2 knockdown. B Colony formation assays in RKO and HCT116 cell lines following TNFR2 knockdown. C Diagram showing the generation of CRC organoid lines from primary CRC tissues (n=3). D Bar graphs showing the relative contributions of point mutation types for selected patients (Tissue, Organoid; n=3 pairs). E Comparative plots of variant classification, variant type, and SNV class between CRC tissues and paired organoids (n=3). F Overview of somatic mutations in key CRC-associated genes, shown for matched tissue–organoid pairs, grouped by patient (T: tissue, O: organoid; n=3 pairs). G Quantification of TNFR2 protein expression (normalized to β-Actin) in CRC organoids with TNFR2 knockdown (n = 3). Supplementary Figure S7. TNFα and TNFR2 inhibitor suppress the progression of CRC (related to Figure 7). A Representative images of tumours from treated mice (n = 5 per group). B Body weight of mice during treatment (n = 5 per group). Arrow indicates the time of treatment initiation.

## Data Availability

The whole-exome sequencing, RNA-seq and Ribo-seq data that support the findings of this study have been deposited in the Genome Sequence Archive [108] in National Genomics Data Center [109], China National Center for Bioinformation/Beijing Institute of Genomics, Chinese Academy of Sciences (GSA-Human: HRA006077) that are publicly accessible at https://ngdc.cncb.ac.cn/gsa-human. The data from human cancer samples were obtained from the TCGA Research Network: http://cancergenome.nih.gov/. The dataset derived from this resource that supports the findings of this study is available at https://xenabrowser.net/datapages/?cohort=TCGA%20Colon%20and%20Rectal%20Cancer%20(COADREAD).

## Abbreviations

CRC: Colorectal cancer
lncRNAs: Long noncoding RNAs
TNFR2: Tumour necrosis factor receptor 2
PDOX: Patient-derived organoid xenograft
EMT: Epithelial–mesenchymal transition
ORFs: Open reading frames
TNFRSF1B: TNF receptor superfamily member 1B
mRNA-seq: mRNA sequencing
RPFs: Ribosome-protected fragments
CDSs: Coding sequences
sORFs: Small open reading frames
TE: Translational efficiencie
UTRs: Untranslated regions
MS: Mass spectrometry
DEGs: Differentially expressed genes
DTEGs: Differential translation efficiency genes
PCA: Principal component analysis
WGCNA: Weighted gene correlation network analysis
GSVA: Gene Set Variation Analysis
GS: Gene significance
MM: Module membership
HCC: Hepatocellular carcinoma
RBP: Retinol-binding protein
qPCR: Quantitative PCR
5'UTR: 5’ untranslated region
uORFs: Upstream open reading frames
AREs: AU-rich elements
IRESs: Internal ribosome entry sites
m^6^A: N6-methyladenosine
WES: Whole exome sequencing
CTCL: Cutaneous T-cell lymphoma
Tregs: Regulatory T cells
STR: Short tandem repeat
ALC: Average local confidence
FDR: False discovery rate
CHX: Cycloheximide
TPM: Transcripts per million
SNV: Single-nucleotide variant
GATK: Genome Analysis Toolkit
GO: Gene Ontology
KEGG: Kyoto Encyclopedia of Genes and Genomes
