# Supplementary material for: Unbiased profiling of translational landscape reveals TNFR2 as a translation-dependent vulnerability in colorectal cancer
Source: J Exp Clin Cancer Res. 2026 Apr 11;45:112. doi: 10.1186/s13046-026-03706-6 (PMC13154585; doi:10.1186/s13046-026-03706-6)
Supplement: Supplementary file 2 — Supplementary Material 2. Supplementary Figure S1. Basic information on active translation and the MHC-I immunopeptidome in CRC (related to Figure 1). A Clinical information of 19 CRC patients for omics analysis. B Dot plot showing the proportion of raw sequence reads mapped to tRNAs, mitochondrial genome (MT), ribosomal RNAs (rRNAs), and canonical genes (“clean”) in human CRC Ribo-seq data. Only cleaned reads were used for further analysis. C Beeswarm plot showing the distribution of ribosome footprint lengths across all 34 samples. D Bar plot showing the percentage of reads mapping to the coding sequence (CDS) and 5'/3' untranslated regions (UTRs) of annotated protein-coding genes. Each line represents one sample. E Bar plot showing P-site positions derived from ribosome footprints across the first 150 nt of annotated ORFs (left), and the percentage of footprints aligning with the primary reading frame (right). F Histograms showing gene expression levels measured by Ribo-seq across all 34 samples. Genes passing the 1 TPM cutoff and identified as actively translated by ribotricer are highlighted in red. G Bar plot showing the number of ORFs identified by ribotricer in each sample. H Summary statistics of peptides in six samples. Peptides are classified as immunogenic (Immun_Y, dark) or non-immunogenic (Immun_N, light), and as canonical (C) or noncanonical (NC). I Left: length distribution of peptides across samples. Right: length distribution of immunogenic vs. non-immunogenic peptides. J Heatmap showing peptide profiles across six samples, including peptide counts for each class. Supplementary Figure S2. Regulation of CDS ORFs in the CRC Ribo-seq dataset (related to Figure 2). A Heatmap showing differentially expressed genes (DEGs) in the TCGA CRC cohort (left) and in the internal CRC cohort (right) (Tumour: n=19; Normal: n=15). B GO plot showing representative Gene Ontology (GO) terms and pathways enriched among DEGs and DTEGs (p < 0.05). Supplementary Figure S3. [file 13046_2026_3706_MOESM2_ESM.docx]

**Supplementary Figure S1.
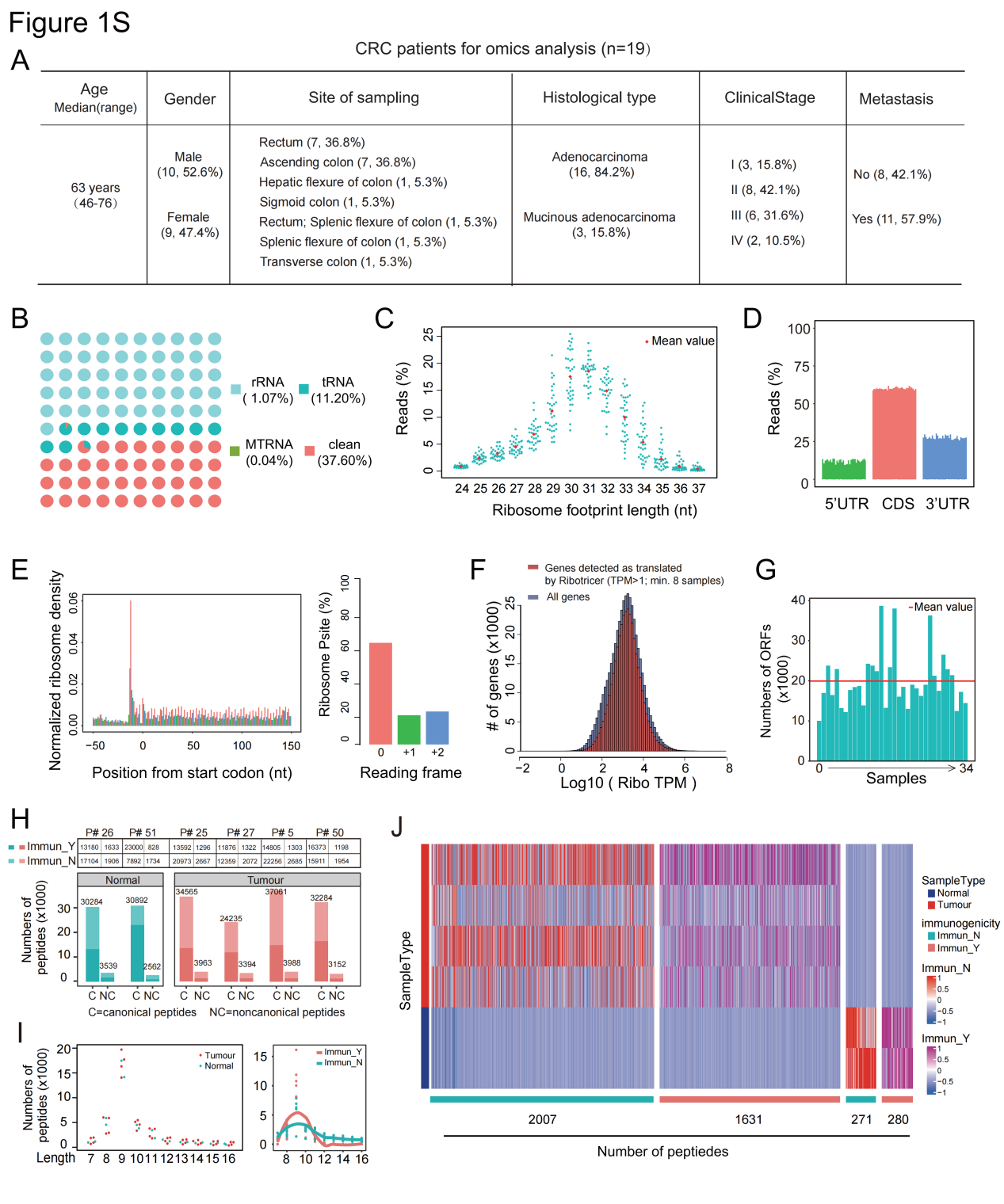
**

**Basic information on active translation and the MHC-I immunopeptidome in CRC (related to Figure 1).** **A** Clinical information of 19 CRC patients for omics analysis**. B** Dot plot showing the proportion of raw sequence reads mapped to tRNAs, mitochondrial genome (MT), ribosomal RNAs (rRNAs), and canonical genes (“clean”) in human CRC Ribo-seq data. Only cleaned reads were used for further analysis. **C** Beeswarm plot showing the distribution of ribosome footprint lengths across all 34 samples. **D** Bar plot showing the percentage of reads mapping to the coding sequence (CDS) and 5'/3' untranslated regions (UTRs) of annotated protein-coding genes. Each line represents one sample. **E** Bar plot showing P-site positions derived from ribosome footprints across the first 150 nt of annotated ORFs (left), and the percentage of footprints aligning with the primary reading frame (right). **F** Histograms showing gene expression levels measured by Ribo-seq across all 34 samples. Genes passing the 1 TPM cutoff and identified as actively translated by ribotricer are highlighted in red. **G** Bar plot showing the number of ORFs identified by ribotricer in each sample. **H** Summary statistics of peptides in six samples. Peptides are classified as immunogenic (Immun_Y, dark) or non-immunogenic (Immun_N, light), and as canonical (C) or noncanonical (NC). **I** Left: length distribution of peptides across samples. Right: length distribution of immunogenic vs. non-immunogenic peptides. **J** Heatmap showing peptide profiles across six samples, including peptide counts for each class.

**
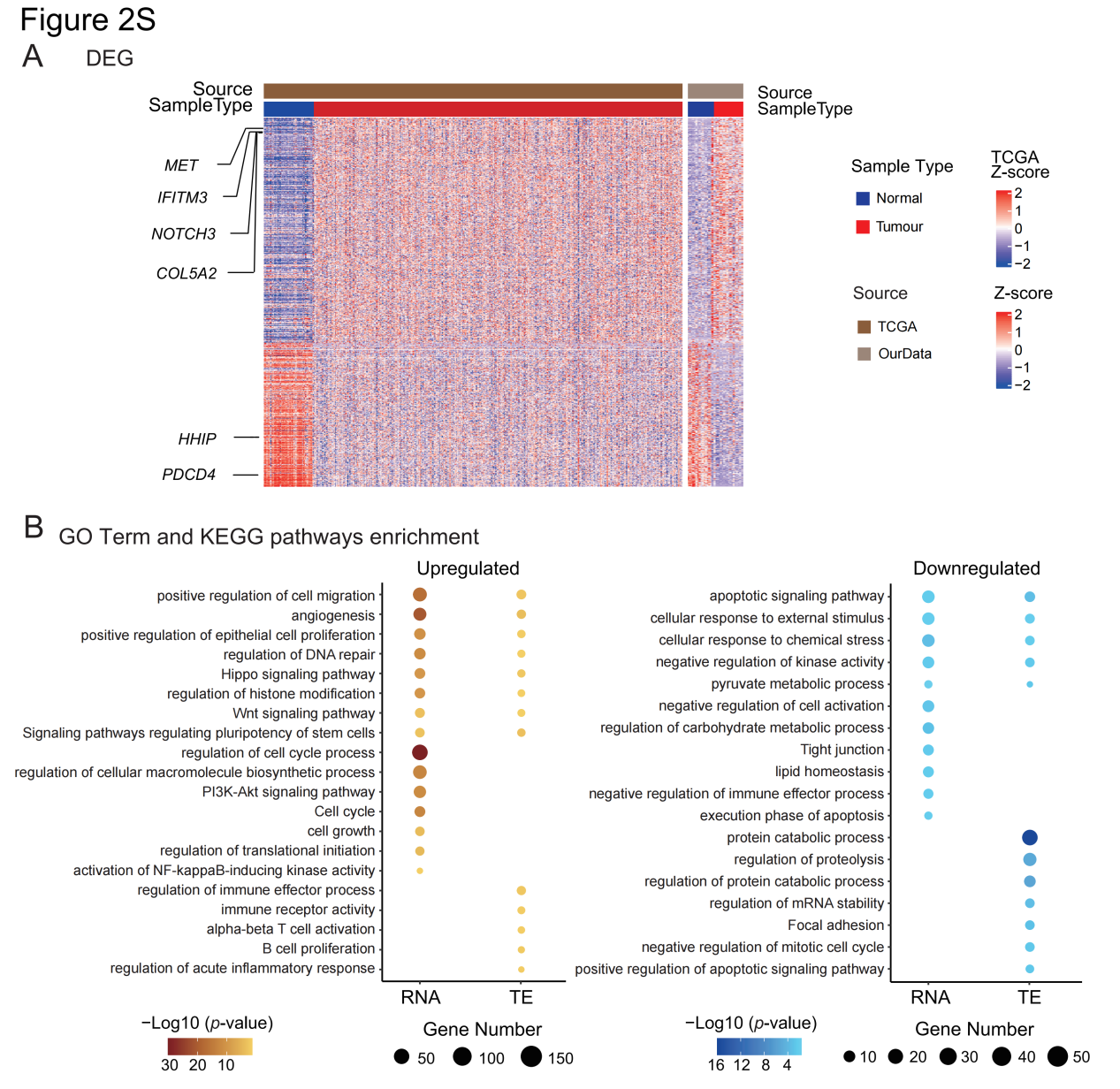
Supplementary Figure S2.**

**Regulation of CDS ORFs in the CRC Ribo-seq dataset (related to Figure 2). A** Heatmap showing differentially expressed genes (DEGs) in the TCGA CRC cohort (left) and in the internal CRC cohort (right) (Tumour: n=19; Normal: n=15). **B** GO plot showing representative Gene Ontology (GO) terms and pathways enriched among DEGs and DTEGs (*p* < 0.05).

**
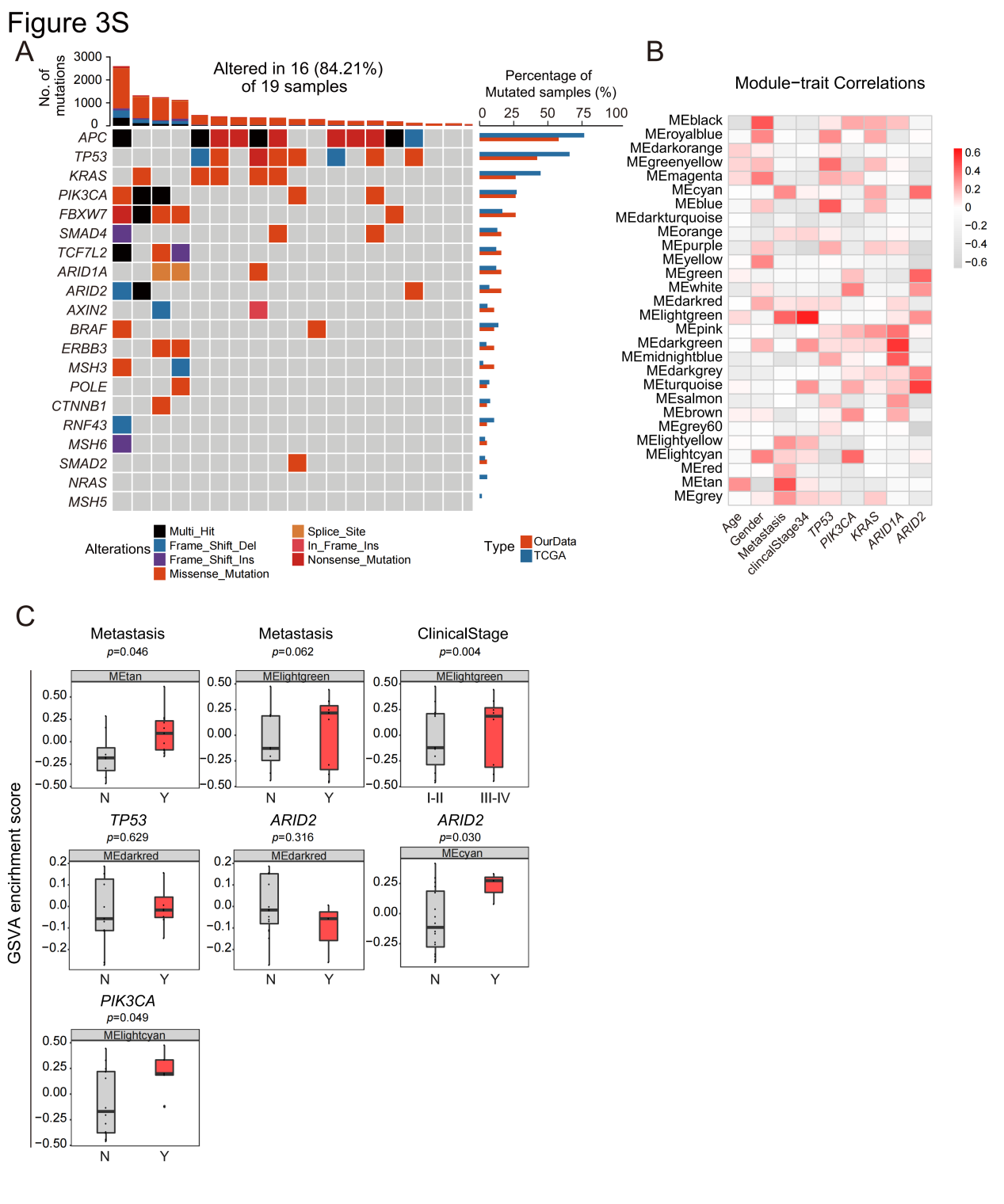
Supplementary Figure S3.**

**Somatic mutational profile of 19 CRC patients and GSVA-based validation of WGCNA module-trait associations (related to Figure 3). A** Oncoplot displaying the most commonly mutated genes in tumour tissues from 19 CRC patients. Samples are ordered by total mutation count. Mutation frequencies in this cohort are compared with those in the TCGA CRC cohort (right panel). **B** Heatmap showing the correlations between WGCNA-identified modules and their corresponding clinical traits or gene mutations, as re-evaluated by GSVA. **C** Box plots showing the GSVA enrichment scores for each significant WGCNA-identified module across sample groups (e.g., metastasis: Y vs N).

**
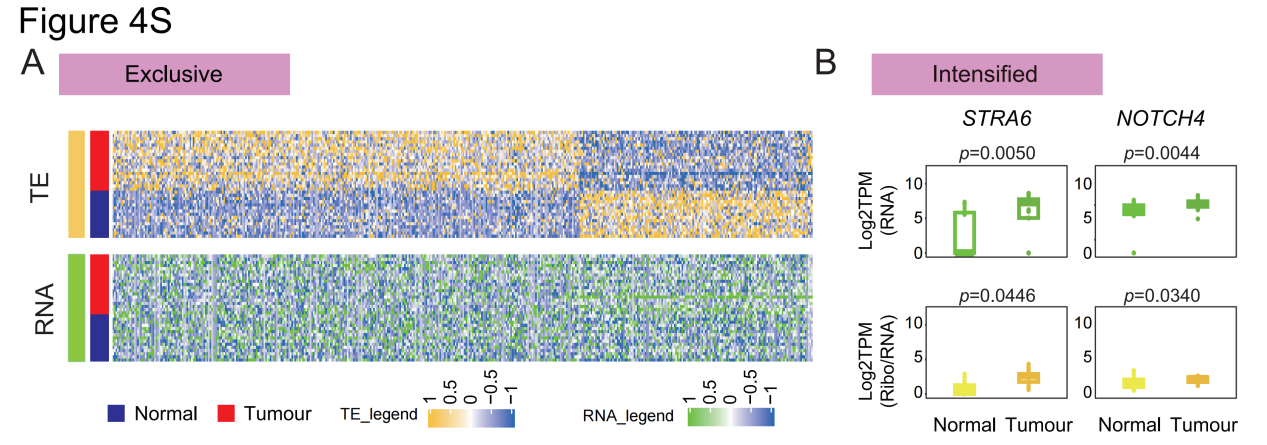
Supplementary Figure S4.**

**Differential gene expression specifically associated with CRC** **(related to Figure 4). A** Heatmap showing different translation efficiency genes (DTEGs) in the Exclusive class (Tumour: n=19; Normal: n=15). **B** Box plots showing expression changes of representative genes in the Intensified class.

**
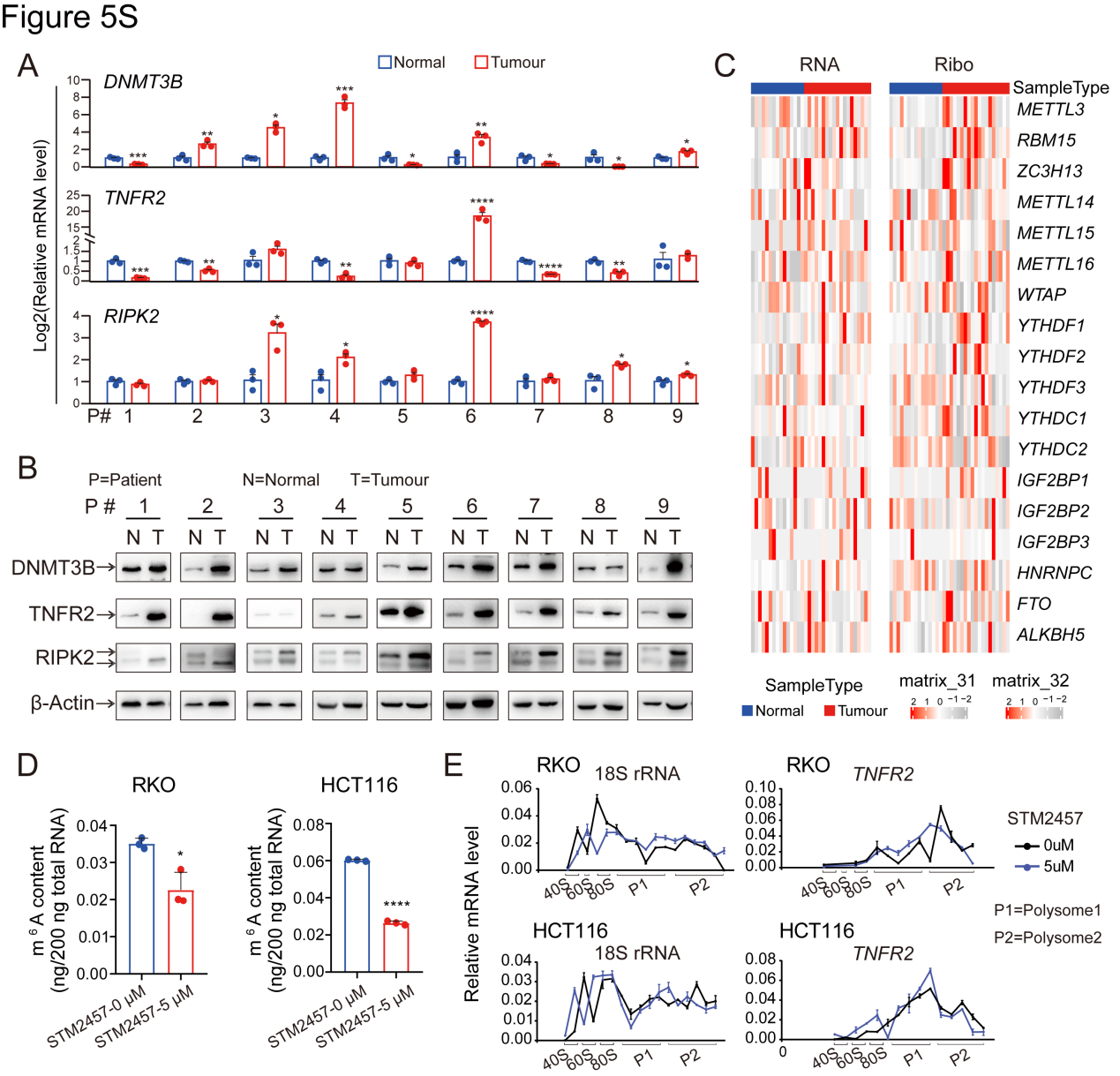
Supplementary Figure S5.**

**Expression analysis of candidate genes and m^6^A-related genes in tissues (related to Figure 5). A** Relative mRNA expression levels of DNMT3B, TNFR2, and RIPK2 in nine paired tumour and paracancerous tissue samples (Normal, Tumour; n=9 pairs). **B** Western blot analysis of DNMT3B, TNFR2, and RIPK2 protein expression in the same paired tissue samples. **C** Heatmap showing the expression of m^6^A-related genes at the transcript and translational levels (Tumour: n=19; Normal: n=15). **D** Detection of m^6^A content per 200 ng of total RNA in RKO and HCT116 cell lines after SIM2457 (5 µM, 48 hours) or DMSO (vehicle) treatment. **E** Polysome qPCR analysis of *TNFR2* expression in RKO and HCT116 cells after SIM2457 (5 µM, 48 hours) or DMSO (vehicle) treatment; 18S rRNA was used as an internal control.

**
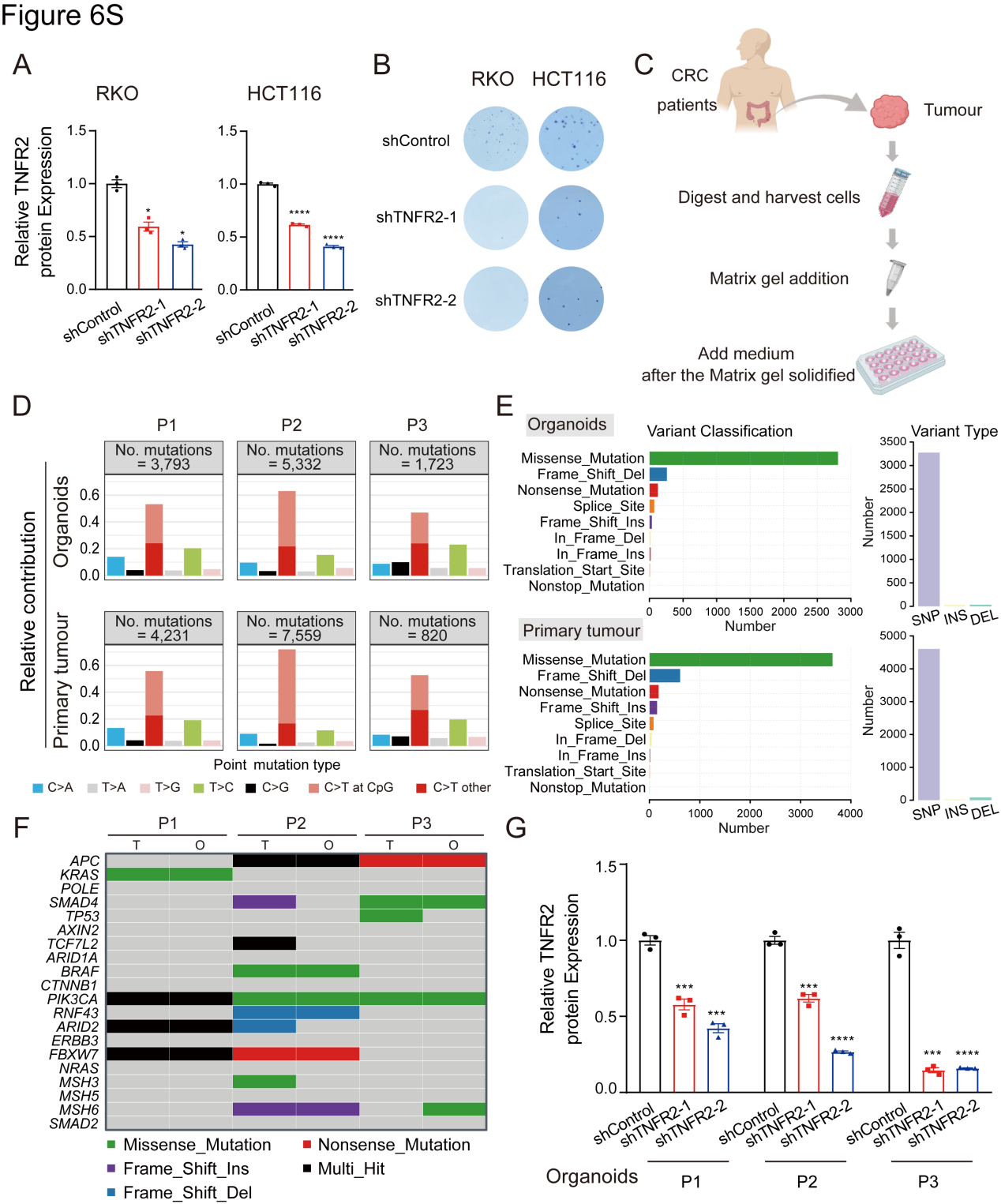
Supplementary Figure S6.**

**Tumour-derived organoids faithfully retain the histological architecture and genomic characteristics of the primary tissues (related to Figure 6). A** Quantification of TNFR2 protein expression (normalized to β-Actin) in RKO and HCT116 cells upon TNFR2 knockdown. **B** Colony formation assays in RKO and HCT116 cell lines following TNFR2 knockdown. **C** Diagram showing the generation of CRC organoid lines from primary CRC tissues (n=3). **D** Bar graphs showing the relative contributions of point mutation types for selected patients (Tissue, Organoid; n=3 pairs). **E** Comparative plots of variant classification, variant type, and SNV class between CRC tissues and paired organoids (n=3). **F** Overview of somatic mutations in key CRC-associated genes, shown for matched tissue–organoid pairs, grouped by patient (T: tissue, O: organoid; n=3 pairs). **G** Quantification of TNFR2 protein expression (normalized to β-Actin) in CRC organoids with TNFR2 knockdown (n = 3).

**
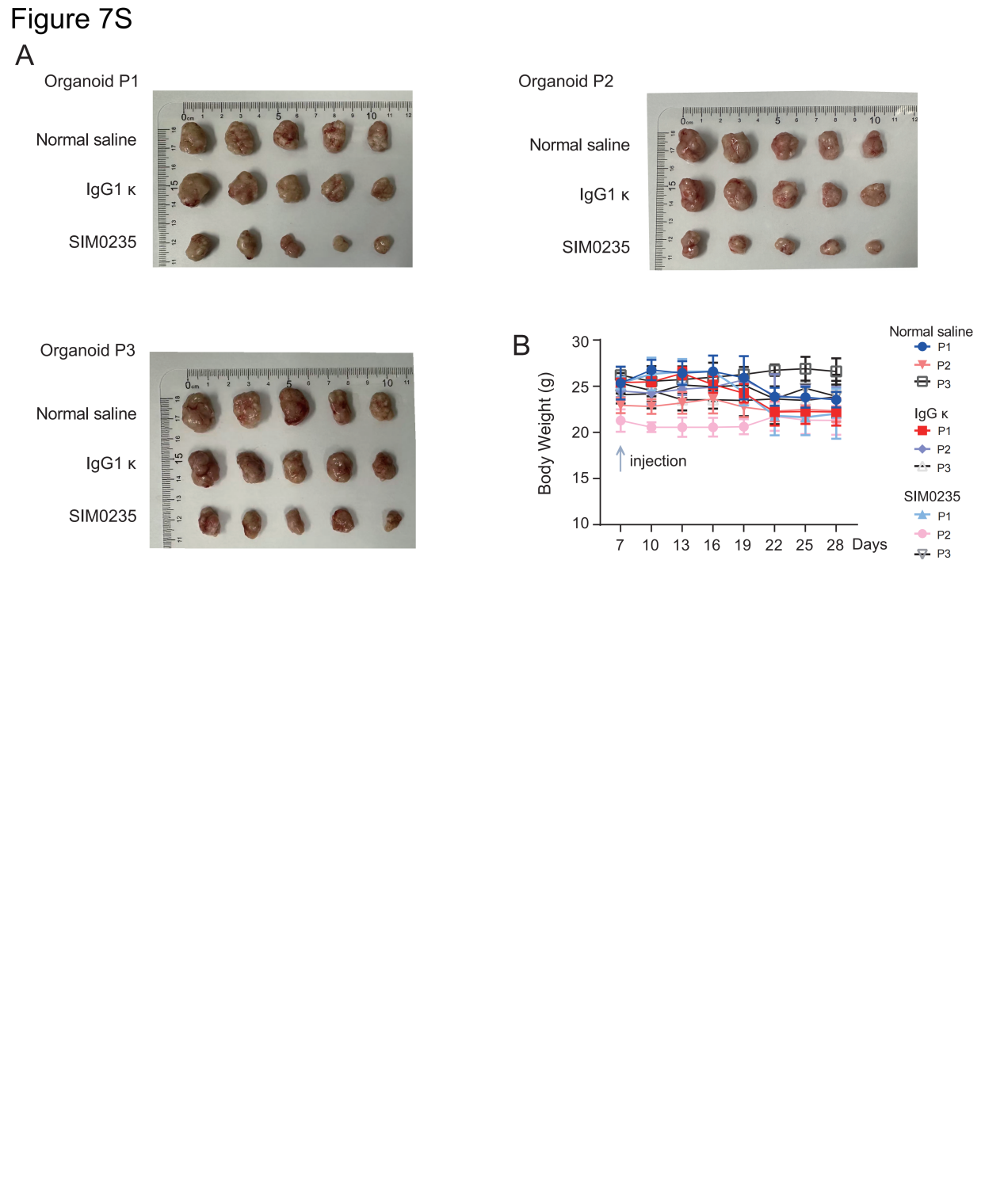
Supplementary Figure S7.**

**TNFα and TNFR2 inhibitor suppress the progression of CRC (related to Figure 7).** **A** Representative images of tumours from treated mice (n = 5 per group). **B** Body weight of mice during treatment (n = 5 per group). Arrow indicates the time of treatment initiation.
